# Structure–Activity Relationship Analysis of Benzimidazoles as Emerging Anti-Inflammatory Agents: An Overview

**DOI:** 10.3390/ph14070663

**Published:** 2021-07-11

**Authors:** Ravichandran Veerasamy, Anitha Roy, Rohini Karunakaran, Harish Rajak

**Affiliations:** 1 Pharmaceutical Chemistry Unit, Faculty of Pharmacy, AIMST University, Semeling 08100, Kedah, Malaysia; 2 Saveetha Dental College and Hospitals, Saveetha Institute of Medical and Technical Sciences, Chennai 600077, Tamil Nadu, India; 3 Department of Pharmacology, Saveetha Dental College and Hospitals, Saveetha Institute of Medical and Technical Sciences, Chennai 600077, Tamil Nadu, India; anitharoy2015@gmail.com; 4 Faculty of Medicine, AIMST University, Semeling 08100, Kedah, Malaysia; rohini@aimst.edu.my; 5 SLT Institute of Pharmaceutical Sciences, Guru Ghasidas University, Bilaspur 495009, India; harishdops@yahoo.co.in

**Keywords:** benzimidazole, cyclooxygenase, bradykinin, cannabinoid, effect of structural modification

## Abstract

A significant number of the anti-inflammatory drugs currently in use are becoming obsolete. These are exceptionally hazardous for long-term use because of their possible unfavourable impacts. Subsequently, in the ebb-and-flow decade, analysts and researchers are engaged in developing new anti-inflammatory drugs, and many such agents are in the later phases of clinical trials. Molecules with heterocyclic nuclei are similar to various natural antecedents, thus acquiring immense consideration from scientific experts and researchers. The arguably most adaptable heterocyclic cores are benzimidazoles containing nitrogen in a bicyclic scaffold. Numerous benzimidazole drugs are broadly used in the treatment of numerous diseases, showing promising therapeutic potential. Benzimidazole derivatives exert anti-inflammatory effects mainly by interacting with transient receptor potential vanilloid-1, cannabinoid receptors, bradykinin receptors, specific cytokines, 5-lipoxygenase activating protein and cyclooxygenase. Literature on structure–activity relationship (SAR) and investigations of benzimidazoles highlight that the substituent’s tendency and position on the benzimidazole ring significantly contribute to the anti-inflammatory activity. Reported SAR analyses indicate that substitution at the N1, C2, C5 and C6 positions of the benzimidazole scaffold greatly influence the anti-inflammatory activity. For example, benzimidazole substituted with anacardic acid on C2 inhibits COX-2, and 5-carboxamide or sulfamoyl or sulfonyl benzimidazole antagonises the cannabinoid receptor, whereas the C2 diarylamine and C3 carboxamide substitution of the benzimidazole scaffold result in antagonism of the bradykinin receptor. In this review, we examine the insights regarding the SARs of anti-inflammatory benzimidazole compounds, which will be helpful for researchers in designing and developing potential anti-inflammatory drugs to target inflammation-promoting enzymes.

## 1. Introduction

**Figure 1:**
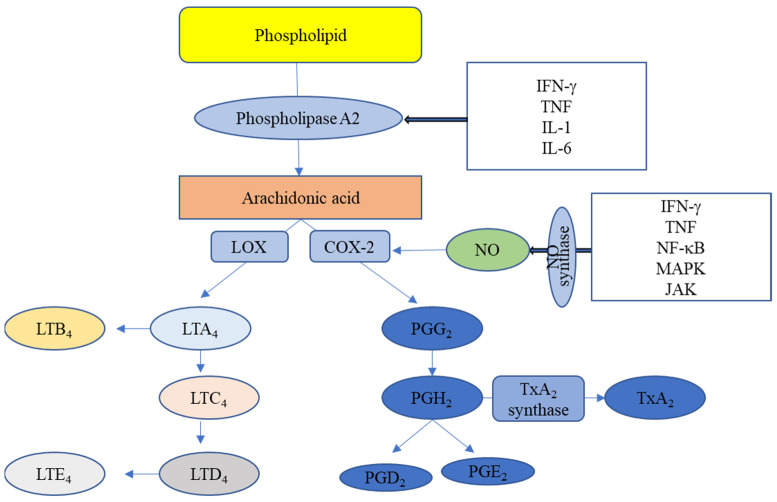
The biochemical process of inflammation. COX—cyclooxygenase; LOX—lipoxygenase; PG—prostaglandin; LT—leukotriene; Tx—thromboxane; NO—nitric oxide; IFN-γ—interferon; TNF—tumour necrosis factor; NF-κB—nuclear factor-κB; MAPK—mitogen activated protein kinase; JAK—Janus kinase; IL—interleukin.

Inflammation is derived from the Latin word “inflammare”. The body’s immune system initiates an immediate response to harmful stimuli, such as infections or any type of irritation [1]. The inflammatory responses entail several biochemical events (Figure 1). They are a defensive attempt by the body to heal infections; however, if inflammation is not controlled, it can prompt a cluster of acute, chronic and systemic inflammatory disorders [2,3]. The major symptoms of inflammation are redness, pain and swelling [4]. Some diseases, such as cardiovascular disease, autoimmune diseases, periodontal disease, Alzheimer’s disease, asthma, diabetes and COPD, are related to chronic inflammation [1,2]. Steroid drugs have traditionally been used to treat inflammation, but their use has gradually decreased due to their adverse effects [5]. Non-steroidal anti-inflammatory drugs have been introduced to overcome the adverse effects of steroidal drugs. The role of cyclooxygenase and its coenzyme in the inflammatory process was an uncreditable discovery [6,7]. In recent years, elucidating the various complex mechanisms behind the inflammatory process has indicated new methods for its treatment [8,9].

Over 75% of the medications currently used have heterocyclics containing nitrogen, oxygen or sulphur, and nitrogen heterocyclics are present in various therapeutically active compounds [10,11]. Pyrazole/pyrazoline, benzimidazole, indole and pyrimidine are important nitrogen-containing heterocyclics in anti-inflammatory research [12]. 

**Figure 2:**
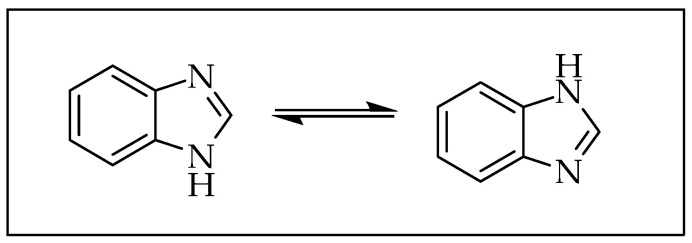
Structure of benzimidazole.

**Figure 3:**
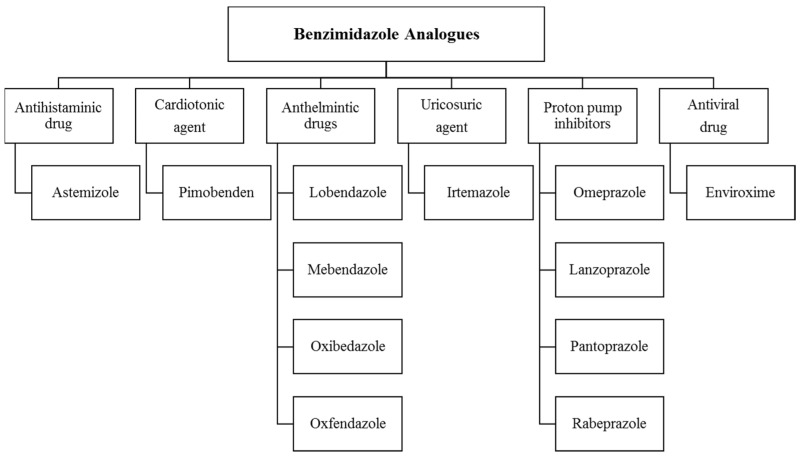
Clinically approved drugs with a benzimidazole nucleus.

Benzimidazole is bicyclic, comprising a benzene fused with an imidazole ring, a heteroaromatic compound with an amphoteric property (Figure 2). This privileged scaffold exhibits anti-convulsant, antioxidant, anti-microbial, anticancer, anthelmintic, anti-inflammatory, anti-fungal, antiviral, antipsychotic and antihistaminic effects, among others [13]. Research on the benzimidazole nucleus has resulted in drugs such as albendazole, mebendazole, thiabendazole, omeprazole, lansoprazole, pantoprazole, astemizole, enviroxime, candesartan, cilexitil, telmisartan and numerous other compounds for treating other diseases (Figure 3) [14].

**Figure 4:**
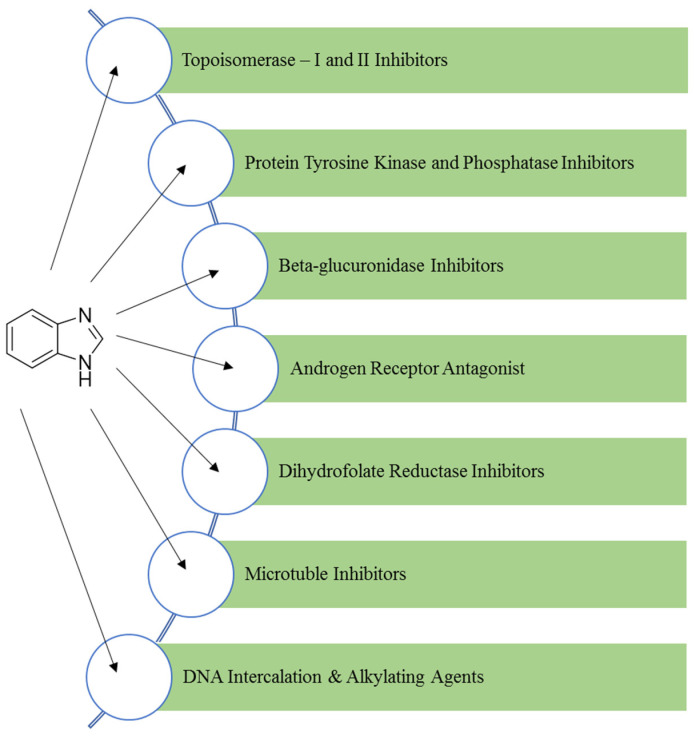
Various biological targets for benzimidazole.

The various targets for benzimidazole are shown in Figure 4. The NH group of benzimidazole is strongly acidic as well as weakly basic in nature. The ionisation constant (pKa) of benzimidazole is 12.8, and its conjugate acid is 5.6 [15]. 

**Figure 5:**
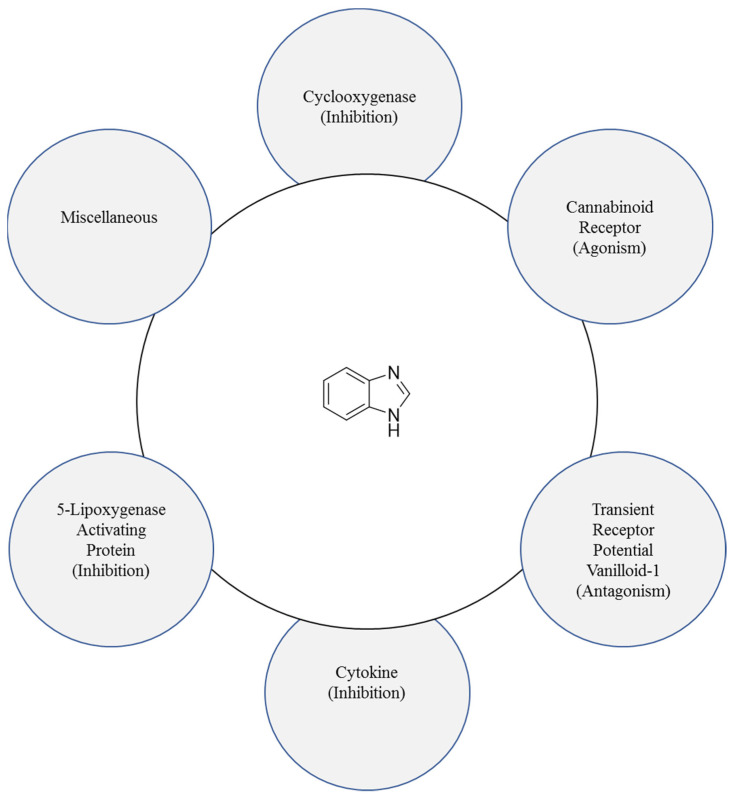
Benzimidazole’s interactions with clinically approved targets.

Benzimidazoles perform their anti-inflammatory activity mainly by interacting with transient receptor potential vanilloid-1, cannabinoid receptors, bradykinin receptors, specific cytokines and 5-lipoxygenase activating protein and cyclooxygenase (COX) (Figure 5).

Even though benzimidazole derivatives are widely used to treat various diseases, including inflammation, they show some side effects, low potential and physicochemical problems. Therefore, discovering new, safer and more potent anti-inflammatory benzimidazoles with reduced side effects is urgently warranted. In recent decades, there have been various reports on the anti-inflammatory activity of benzimidazoles. Hence, the goal of this review was to collect the existing data related to the anti-inflammatory activity of benzimidazoles and assess the obtained results, thus facilitating the discussion of structure–activity relationships (SARs). 

## 2. Method

A literature search was carried out using the Web of Science citation indexing, PubMed and Google Scholar by using the terms “Heterocyclic”, “Benzimidazole”, “Structure activity relationship” and “Anti-inflammatory” in combination with the words “cyclooxygenase”, “cannabinoid”, “cytokine” or “lipoxygenase”, finding thousands of publications. Additionally, the terms “Benzimidazole” and “Anti-inflammation action” were searched alone, and the results were reduced to 75 hits. The publications were reviewed by title, abstract and text and reduced to those dealing with benzimidazoles with anti-inflammatory action and their structure–activity relationships (SARs). Furthermore, studies without structural and proper pharmacological study information were excluded. The data from the remaining publications were collected and categorised according to the actions of benzimidazoles on different enzymes responsible for inflammation. 

## 3. Structure–Activity Relationships of Benzimidazole Derivatives as Anti-Inflammatory Agents

The reported SAR analysis indicates that substitutions at the N1, C2, C5 and C6 positions of the benzimidazole nucleus greatly influence the anti-inflammatory activity. Various heterocycle substitutions at N1 of benzimidazole account for the effective anti-inflammatory effects of various agents. 

### 3.1. Cyclooxygenase Inhibitors

Cyclooxygenase (COX) is a crucial enzyme in the biosynthesis of prostaglandins and thromboxanes. COX-1 is a constitutive enzyme produced in many tissues, while COX-2 is inducible and is expressed during inflammation. Hence, drugs that inhibit COX-2 are better anti-inflammatory agents.

**Figure 6:**
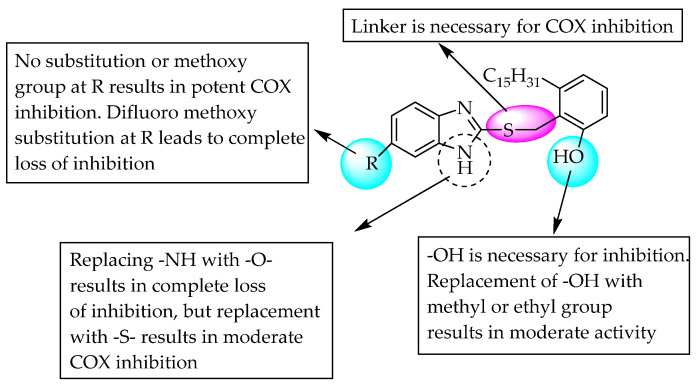
Structure-Activity Relationships (SARs) of anacardic acid-conjugated benzimidazole derivatives.

Paramashivappa et al., in the year 2003, prepared 2-[[2-alkoxy-6-pentadecyl-phenyl)methyl]thio]-1*H*-benzimidazoles by incorporating benzimidazole with anacardic acid and evaluated its COX-2-inhibitory activity in humans (Figure 6). A compound with R = H and R1 = methoxy moiety showed potent anti-inflammatory activity, with 384-fold selectivity for COX-2 over COX-1 inhibition. Meanwhile, the other compound with R = methyl and R1 = H moiety showed 470-fold selectivity, comparable to the 375-fold and 200-fold selectivity of celecoxib and rofecoxib, respectively. The study also confirmed the importance of the “NH” moiety of benzimidazole in anti-inflammatory activity. However, substitution of –CH_3_ or –NO_2_ at C5 of benzimidazole showed moderate inhibition, whereas –OCHF_2_ did not show favourable inhibitory action [16].

**Figure 7:**
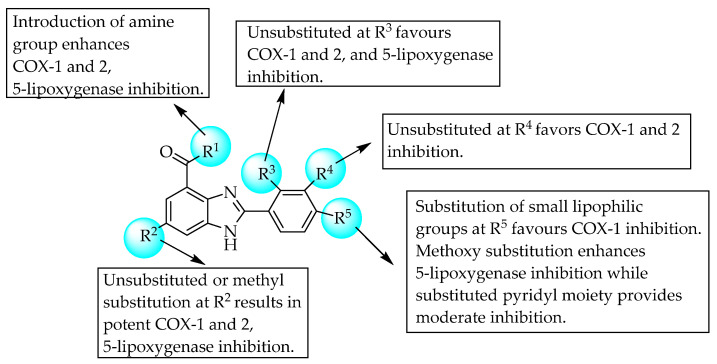
SARs of 2-phenyl-substituted benzimidazoles.

In 2016, Bukhari et al. studied COX and 5-lipoxygenase inhibition by a few 2-phenyl-substituted benzimidazoles. As shown in Figure 7, unsubstituted at R^2^, R^3^ and R^4^ is preferred for COX-1 and 2, and 5-lipoxygenase inhibition, whereas the amine group at R^1^ enhanced the inhibition of all three enzymes. However, the lipophilic group at R^5^ favours COX-1 inhibition, a hydrophilic group enhances COX-2 inhibition and a methoxy substitution favours 5-lipoxygenase inhibition. One of the compounds with R^2^–CH_3_, R^1^–NH_2_ and R^3^ and R^4^–H showed better COX-1 inhibition, with IC_50_ = 0.72 ± 0.77 µM. However, another compound bearing a nitrile group at position R^5^ was a dual (COX-1 and -2) inhibitor with IC_50_s of 8.17 ± 2.85 and 6.79 ± 1.46 µM, respectively. Another compound, having 2-aminopyridin-4-yl at R^5^, showed better 5-lipoxygenase inhibition, with an IC_50_ of 8.41 ± 1.22 µM. Overall, the substitution of recommended groups at the above-mentioned position favours potent COX-1 and 2, and 5-lipoxygenase inhibition [17]. The SAR of the compounds is shown in Figure 7.

### 3.2. Cannabinoid Receptor Agonist

Cannabinoid receptor agonists are effective for the treatment of inflammation. These effects are mediated via two subtypes of cannabinoid receptors: CB1, located centrally and peripherally, and CB2, on immune cells or peripheral tissues. 

**Figure 8:**
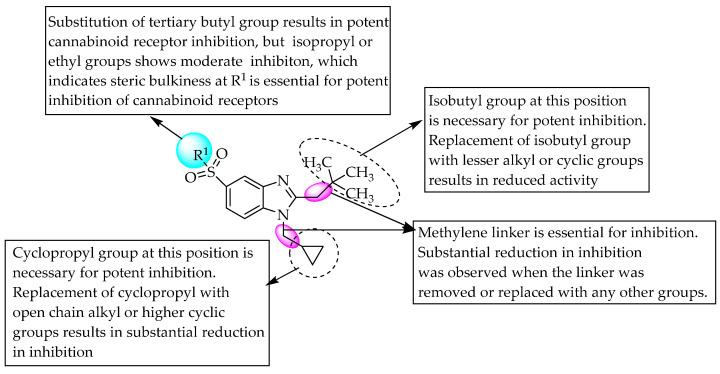
SARs of 5-sulfamoyl substituted benzimidazoles.

Scientists from AstraZeneca reported cannabinoid receptor agonist, CB2 agonist and CB1/CB2 dual agonist activity for benzimidazole derivatives as selective for the management of pain and inflammation. A compound having a carboxamide substitution at C5 of benzimidazole was a highly selective CB2 agonist, with 970-fold selectivity over CB1 receptors. It also showed Ki values of 3170 and 3.3 nM for the human CB1 and CB2 receptors, respectively. The replacement of the carboxamido group in the previous compound with the sulfamoyl group produced another comparable cannabinoid receptor agonist (Figure 8). 

Furthermore, an AstraZeneca scientist also studied the CB1/CB2 dual agonist activity of benzimidazoles with a large polar 5-*N*-sulfonamide substituent for the management of pain [18]. In 2011, scientists from Pfizer reported CNS-penetrant, selective CB2 agonist activity for a few sulfonyl benzimidazole derivatives as potential analgesic and anti-inflammatory agents with fewer side effects [19]. A tertiary butyl substituent at R^1^ resulted in potent cannabinoid receptor antagonism. However, a methyl linker between C2 of benzimidazole and an isobutyl group, and benzimidazole N1 and a cyclopropyl group, is essential for potent agonistic activity (Figure 8).

**Figure 9:**
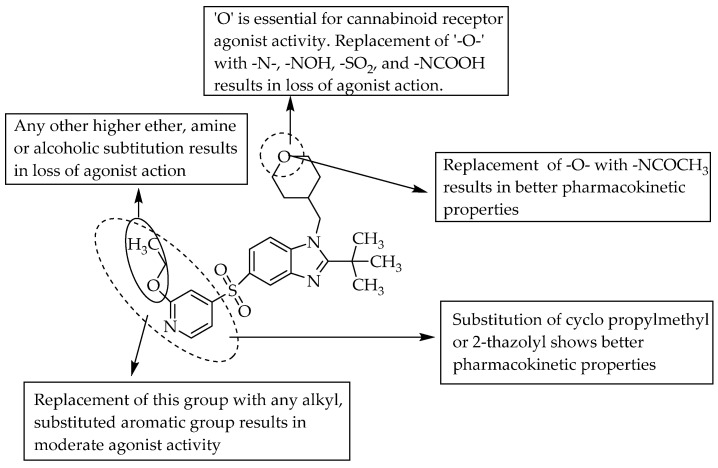
SARs of 2-isbutyl-5-sulfonylbenzimidazole derivatives.

Gijsen and co-workers, in 2012, evaluated the CB2-receptor agonist activity of 5-sulfonyl benzimidazole derivatives. One of the compounds having a 2-ethoxypyridin- 4-yl sulfonyl moiety substitution at C5 of benzimidazole showed good selectivity, with a decent drug-like profile. Further studies by the same research group led to the development of two potent compounds with methyl-cyclopropyl and thiazole moieties, respectively, made by replacing the 2-ethoxypyrimidin-4yl group in the compound shown in Figure 9 [20].

### 3.3. Bradykinin Receptor Antagonists

Several acute and chronic inflammatory pathways resulting in pain, oedema and vasodilation are aggravated by kinins, bradykinin and kallidin [21,22,23]. G-protein-coupled receptors for bradykinins B1 and B2 mediate these effects [24]. In most cell types, the bradykinin B2 receptor is expressed under normal conditions, whereas the bradykinin B1 receptor is expressed in infections, inflammatory diseases and traumatic tissue injury.

**Figure 10:**
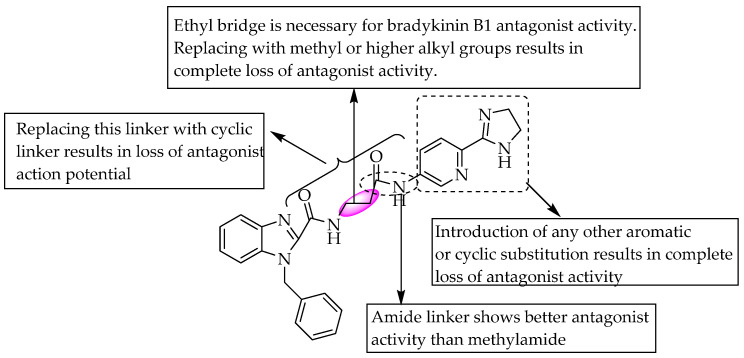
SARs of 2-substituted N-benzyl benzimidazoles.

Guo et al., in 2008, introduced a benzimidazole nucleus into a benzodiazepine derivative with potent bradykinin B1 receptor antagonistic activity, by replacing the phenethyl benzodiazepine moiety, and reported anti-inflammatory activity. Their study showed a potent antagonistic effect at bradykinin B1 receptors with better bioavailability. The combination of the β-alanine linker and 2-imidazoline-5-aminopyridine group at the C2 of benzimidazole resulted in excellent potency, with IC_50_ = 2 nM. They also revealed that the 2-carboxamide group on benzimidazole was necessary for the activity. Still, the activity was reduced when the ethyl linker between the two carboxamide groups was replaced with a methyl or any other longer alkyl group [25] (Figure 10). They concluded that the hydrogen bond acceptor in these compounds seemed to enhance the effect.

**Figure 11:**
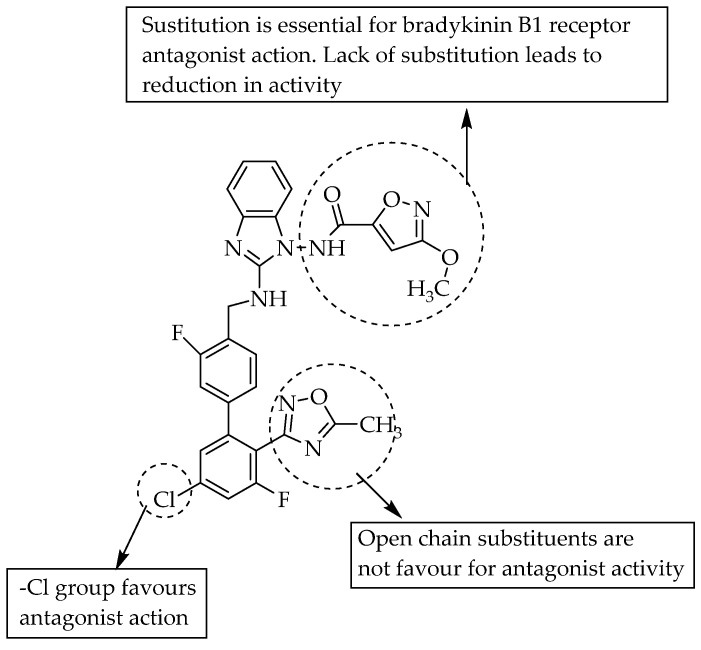
SARs of 2-aminobenzimidazole as bradykinin B1 receptor antagonists.

As in a previous study, Zischinsky et al. (2010) also studied the bradykinin B1 receptor antagonist activity of small benzimidazole derivatives (Figure 11). They found a compound containing an acetamide moiety with better activity, with IC_50_ = 15 nM, than the parent compound, with an IC_50_ value of 3500 nM. They performed further optimisation in the substituted moieties and obtained a compound with IC_50_ = 0.7 nM. A chloroimidazole derivative with IC_50_ = 0.3 nM was the most active compound in their study [26].

### 3.4. Anticytokines

Anticytokine therapies are used in the treatment of chronic inflammatory diseases, particularly autoimmune diseases such as rheumatoid arthritis. Based on the first introduced principle of cytokine blockade in the 1990s, tumour necrosis factor (TNF)-α antagonists still represents the leading anti-cytokine therapy.

**Figure 12:**
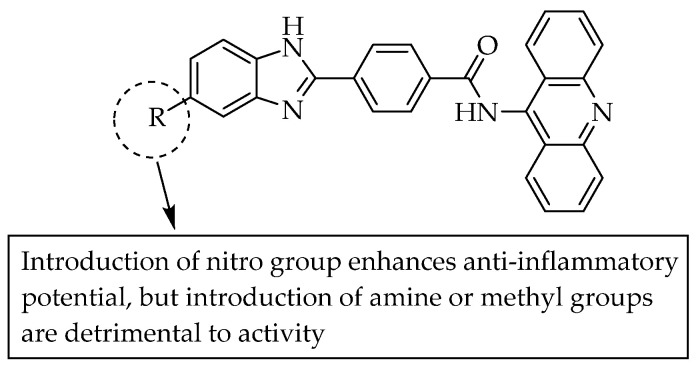
SARs of benzimidazole-acridine derivatives.

A number of *N*-acridin-9-yl-4-benzimidazo-2-ylbenzamides (Figure 12) were synthesised and screened for anti-inflammatory and CDK-inhibitory activities. The study revealed that the substitution at C5 of benzimidazole plays a crucial role in anti-inflammatory and CDK-inhibitory activities. Pronounced activity against CDK1 and CDK5 was shown by a compound bearing a nitro group at C5. By contrast, an amino or methyl group at C5 led to the complete loss of the anti-inflammatory and CDK-inhibitory activity [21]. 

**Figure 13:**
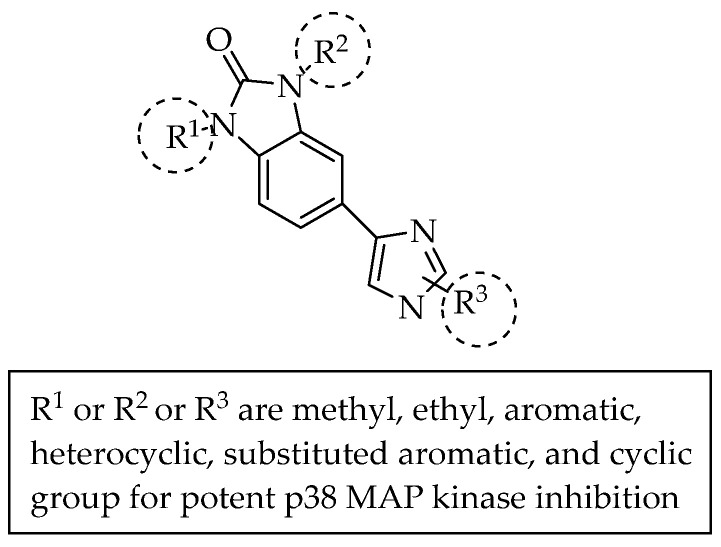
Benzimidazol-2-one compounds bearing five positions with substituted imidazole.

**Figure 14:**
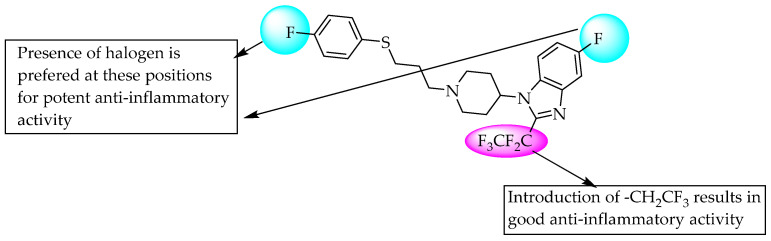
SARs of 1,2,5-trisubstituted benzimidazole as CCR3 receptor inhibitor.

A series of 550 benzimidazol-2-one compounds bearing five positions with substituted 5-membered heteroaryls containing one to two heteroatoms of nitrogen, sulphur or oxygen (Figure 13) was synthesised by Dombroski et al. (2006) and found to be potent inhibitors of p38 MAP kinase [22]. In 2007, Anderskewitz et al. reported that the compound shown in Figure 14 inhibited the CCR3 receptor, with a binding constant (Ki) of 100 nM. However, substituting the C2-position of benzimidazole with trifluoroethane resulted in better CCR3 receptor inhibition, with Ki = 19 nM [23]. 

**Figure 15:**
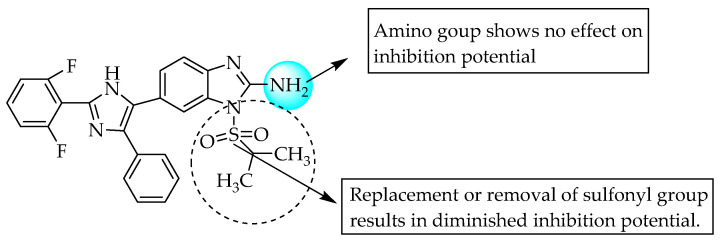
SAR of 2-amino-1-isopropylsulfonyl 6-substituted benzimidazole.

**Figure 16:**
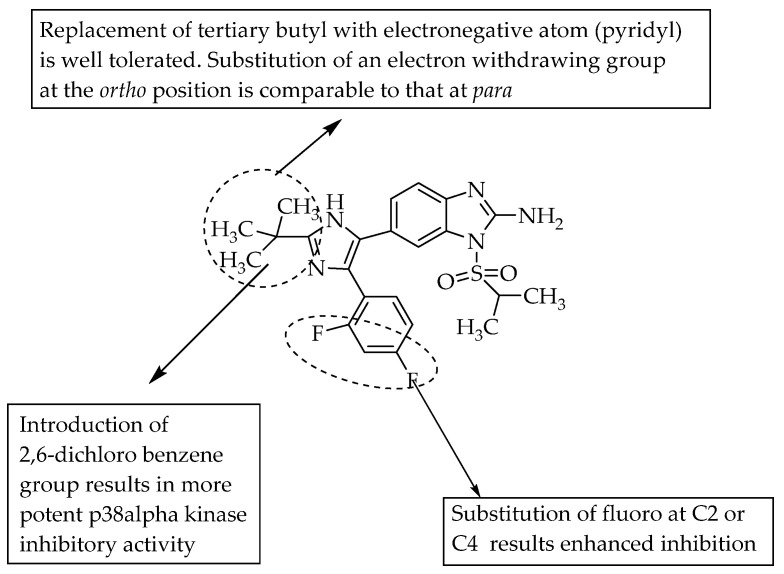
SARs of 2-amino-1-isopropylsulfonyl 6-substituted benzimidazole.

Mader et al. (2008) introduced 2-amino-1-isopropylsulfonyl 6-substituted benzimidazole (Figure 15 and Figure 16) as a potent TNF-α and p38α MAP kinase inhibitor. A 2,6-dichloro or difluoro phenyl moiety at the 2-position of imidazole substituted at C6 of benzimidazole enhanced the inhibition of TNF-α and p38α MAP kinase activity. However, 2- and 4-fluoro phenyl moieties at the 4-position of imidazole substituted at C6 of benzimidazole enhanced the inhibition potential for TNF-α and p38α MAP kinase activity [24].

**Figure 17:**
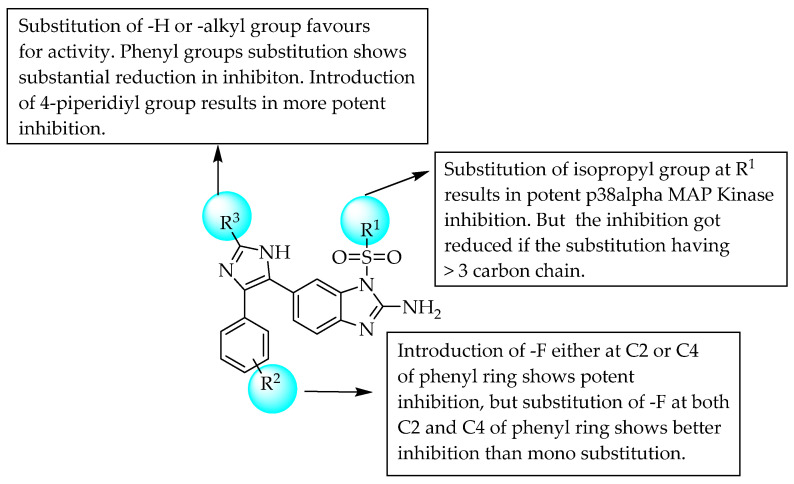
SARs of sulfonyl group incorporated 2-aminobenzimidazoles.

In 2005, Dios et al. studied the importance of the *N*-sulfonyl group incorporated 2-aminobenzimidazole as a p38α MAP kinase inhibitor. The compound with a piprid-4-yl at R^3^ showed the highest efficacy and selectivity. A 2,4-difluoro substitution at R^2^ led to better inhibition than mono-fluoro substitution; moreover, optimum steric bulky groups with three carbons at R^1^ favoured p38α MAP kinase inhibition [27]. However, bulkier groups at R^1^ led to a reduction in the inhibition of p38α MAP kinase activity. The SAR of the compounds is shown in Figure 17.

**Figure 18:**
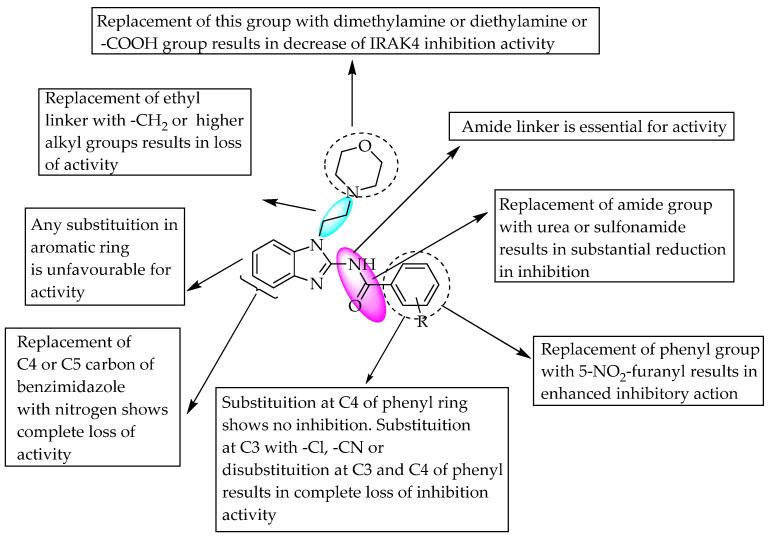
SARs of 2-acetamidopheny-1-(N-substituted) benzimidazoles.

**Figure 19:**
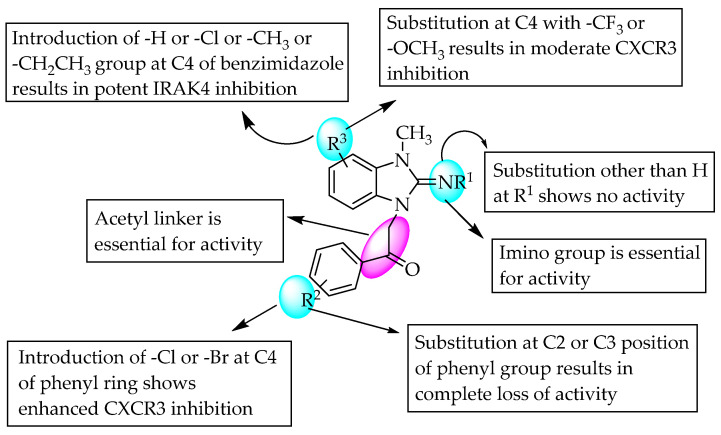
SARs of 3-methyl-1,2-disubstituted benzimidazoles.

Amgen Inc. (South San Francisco, CA, USA) carried out high-throughput screening and SAR studies of small molecules, and they found that compound **1** (R = 3-NO_2_) was a potent inhibitor of interleukin-1 (IL-1) receptor-associated kinase-4 (IRAK4). They also studied the significance of an amide linker at the benzimidazole C2 position by removing the amide carbonyl, which resulted in a substantial reduction in inhibition. Furthermore, their study continued by replacing the amide with urea and sulphonamide groups, which resulted in the loss of IRAK4 inhibition, which indicates the need for an amide bridge between the two aromatic groups for good activity (Figure 18) [28]. As a continuation of the previous research, the Frankel and Powers team obtained a US patent for their study on similar *N*-acyl 2-aminobenzimidazole derivatives containing various aroyl and hetero aroyl substituents at the C2 position as potent IRAK4 inhibitors (Figure 18) [29]. Abbott Corporate also performed high-throughput screening through binding studies to identify potent 1,2-disubstitutedbenzimidazole derivatives as CXCR3 antagonists, inhibiting CXCL10’s binding to CHO cell membranes. A compound having a methyl group at R^3^, chlorine at R^2^ and no substitution at R^1^ (Figure 19) showed maximum CXCR3 antagonism. Furthermore, substitution with a methoxy group at R^3^ did not reduce the activity, but substitutions at the 5- and 6-positions were unfavourable for activity (Figure 19) [30].

**Figure 20:**
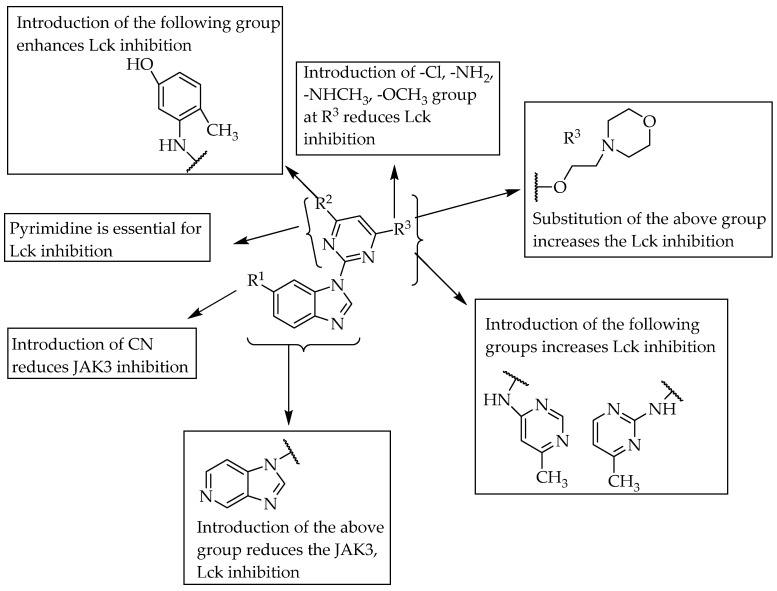
SARs of 2-benzimidazole substituted with pyrimidine.

In 2006, Sabat et al. reported the anti-inflammatory effects of a few benzimidazoles that were 1-substituted with pyrimidin-2-yl. They found that compound **2** (Table 1), by its lymphocyte-specific kinase (Lck) blocking activity, showed a potent anti-inflammatory effect [31]. Compound **2** had an anti-inflammatory effect and was potent at 3 nM in Lck kinase inhibition and potent at 0.054 mM for inhibiting IL-2 cytokine production. They also reported that small group substitutions at R^3^ of the pyrimidine moiety led to a loss of the Lck-inhibitory effect. In the same year (2006), Chen et al. identified compound **3** (Table 1), with a substitution on C6 of benzimidazole, as a potent inhibitor of Janus kinase 3 (JAK3), from their extensive SAR studies [32]. Compound **3** exhibited JAK3-inhibition potency at 45 nM. They found that the substitution of a nitrile group at the 6-position of benzimidazole resulted in excellent JAK3 inhibition. The SARs of the compounds are depicted in Figure 20.

In another study, the activity of pyrimido benzimidazoles in inhibiting lymphocyte-specific protein tyrosine kinase (Lck) was explored by Martin et al. (2008) [33]. The SAR studies revealed that 6-(2,6-dimethyl phenyl)-2-((4-(4-methyl-1-piperazinyl)phenyl) amino) pyrimido [5′,4′:5,6]pyrimido-[1,2-a]benzimidazol-5(6H)-one was the most potent Lck inhibitor, with IC_50_ = 0.007 µM. 

**Figure 21:**
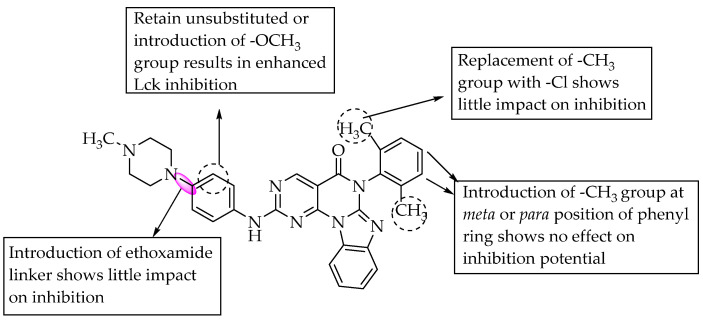
SARs of pyrimido-benzimidazoles.

**Figure 22:**
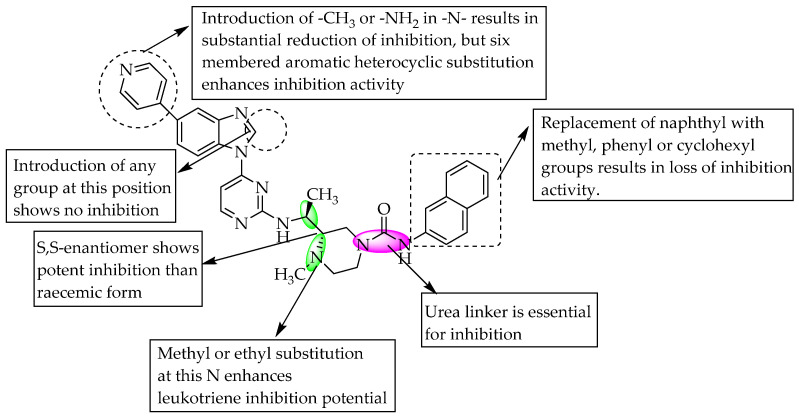
SARs of 4-benzimidazolyl-*N*-piperazinethyl-pyrimidin-2-amines.

Replacing the 2,6-dimethyl phenyl group with chlorine at the 6-position of the fused pyrimidobenzimidazole nucleus resulted in a low impact on activity, but shifting the methyl groups to the meta or para position was unfavourable for Lck inhibition (Figure 21). Hunt et al. (2009) reported potent inhibition of Lck kinase and cellular IL-2 release by 4-benzimidazolyl-*N*-piperazinethyl-pyrimidin-2-amines. The compound shown in Figure 22 inhibited Lck, with IC_50_ = 0.12 nM, and cellular IL-2 release, with IC_50_ = 8 nM [34]. The SARs of the compounds are given in Figure 22. Substitution at the 2-position of benzimidazole was unfavourable for Lck inhibition. Furthermore, the author stated that the S, S enantiomers are more potent than the racemic forms of 4-benzimidazolyl-N-piperazinethyl-pyrimidin-2-amines.

### 3.5. FLAP Inhibitors

Leukotrienes are responsible for initiating and amplifying the inflammatory response by regulating the recruitment and activation of leukocytes in inflamed tissues, and they are lipid mediators. The inhibition of leukotriene biosynthesis may also be achieved by targeting FLAP.

**Figure 23:**
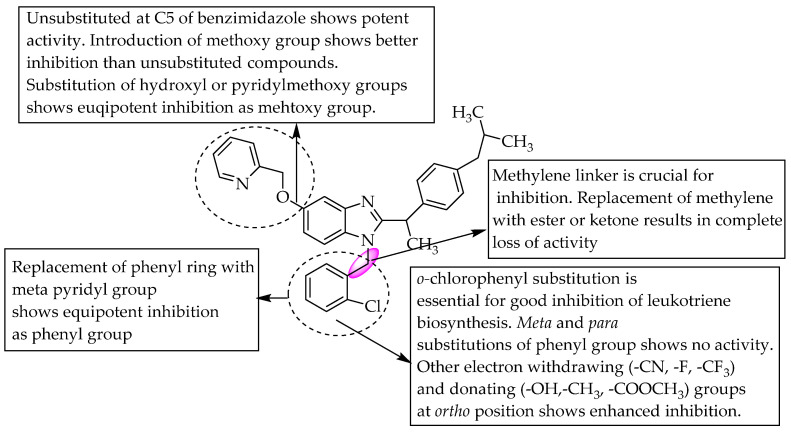
SARs of 2-isobutylphenylethyl benzimidazoles.

Banoglu et al. (2012) examined benzimidazoles as 5-lipoxygenase (5-LO)-activating protein (FLAP) inhibitors. They performed virtual screening using combined ligand- and structure-based pharmacophore modelling to find compounds targeting FLAP. Their study led to the identification of 1-(2-chlorobenzyl)-2-(1-(4-isobutylphenyl)ethyl)-1*H*-benzimidazole, which inhibited leukotriene biosynthesis with IC_50_ = 0.31 mM. In order to optimise the above compound, the authors synthesised a few potent benzimidazole derivatives, with IC_50_ = 0.12–0.19 mM for intact neutrophils [35]. They also stated that no substitution at C5 of benzimidazole, a methyl linker between the N of benzimidazoleand the aryl or heteroaryl groups favoured the inhibition of leukotriene biosynthesis (Figure 23).

AM803, AM643 and AM103 are recently developed FLAP inhibitors that have, preclinically, been shown to be efficacious in inflammatory diseases. They have an indole scaffold that, structurally, is deficient in one nitrogen atom compared to the benzimidazole nucleus [36,37,38,39,40].

### 3.6. TRPV-1 Antagonists

TRPV-1 is a member of the ion channels that allow the transient influx of Ca^2+^ ions when activated and predominantly expressed in peripheral sensory neurons involved in nociception and neurogenic inflammation.

**Figure 24:**
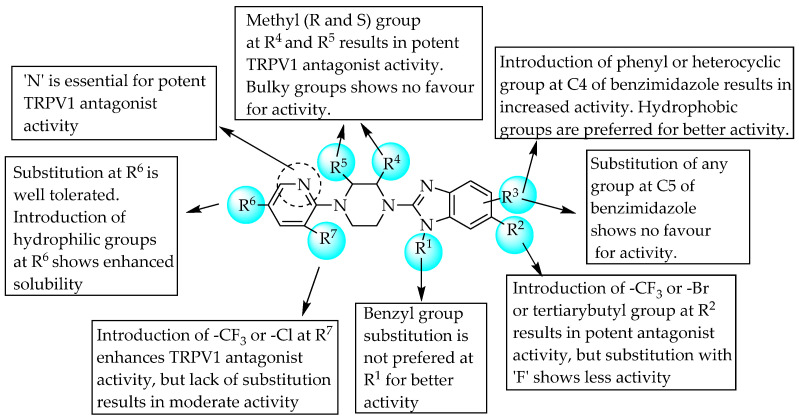
SARs of 2-(4-pyridin-2--ylpiperazin-1-yl)-1*H*-benzo-[d]imidazoles.

Ognyanov et al. (2006) evaluated the TRPV-1-antagonising property of 2-(4-pyridin-2-ylpiperazin-1-yl)-1*H*-benzo-[d]imidazoles, which blocked capsaicin-induced flinch in rats in a dose-dependent manner. One of the compounds, (*R*,*S*)-1-(5-chloro-6- ((*R*)-3-methyl-4-(6-(trifluoromethyl)-4-(3,4,5-trifluorophenyl)-1*H*-benzo[d]imidazol-2-yl)piperazin-1-yl)pyridin-3-yl)ethane-1,2-diol, was the most effective orally bioavailable TRPV-1 antagonist [41]. Conclusively, the simultaneous introduction of bulky lipophilic groups at position 4 of the benzimidazole, a methyl group at R^4^, hydrophilic groups at R^6^ and CF_3_ or Cl at R^7^ was favourable for potent TRPV-1-antagonising activity (Figure 24). Additionally, a patent from Amgen has also demarcated the TRPV-1-antagonising action of a piperazine-linked benzimidazole derivative, WO04035549 [42]. 

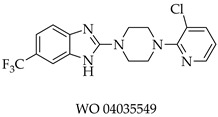



**Figure 25:**
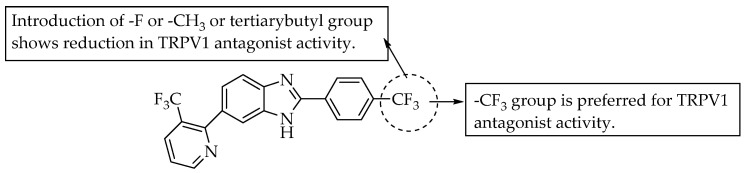
SARs of 6-(3-trifluoromethylpyridin-2-yl) benzimidazoles.

In 2006, Fletcher et al. studied the affinity of compounds containing a para-substituted phenyl at C2 of benzimidazole for the hTRPV-1 receptors (Figure 25). The compound shown in Figure 25 is potent in antagonising hTRPV-1 receptors, with IC_50_ = 22 nM, as measured in a FLIPR-based assay. Moreover, minor changes, such as replacing the 4-CF_3_ group of the phenyl moiety at C2 of benzimidazole with tert-butyl, –CH_3_ or F, led to decreased activity, with IC_50_ values of 224, 170 and 320 nM, respectively [43]. 

### 3.7. Protein Kinase Inhibitors

Protein kinases add a phosphate group to a protein in a process called phosphorylation, which can turn a protein on or off and, therefore, affect its level of activity and function.

**Figure 26:**
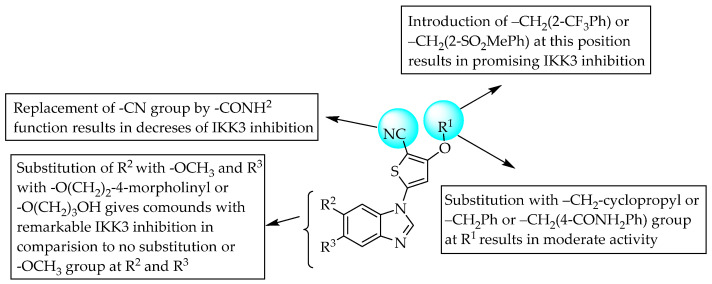
SARs of benzimidazole substituted with thiophene.

In a study, thiophene substitution at position 1 of benzimidazole resulted in a very potent inhibitor of serine–threonine kinase 3 (IKK3). Moreover, the IKK3-inhibitory activity was reduced when one of the hydrogen atoms of R^1^—namely, CH_2_—was replaced with –CH_3_. This indicates that the methylene bridge at R^1^ is essential for potent IKK3 inhibition. However, electronegative groups substituted at the phenyl ring of R^1^ resulted in better IKK3 inhibition than the substitution of electron-donating groups. However, the inhibitory effect on IKK3 was decreased by replacing the nitrile group with an amide group (compound **4**) [44]. The SARs of the compounds are shown in Figure 26.

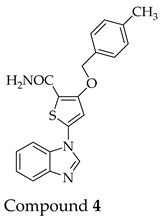



### 3.8. Miscellaneous

**Figure 27:**
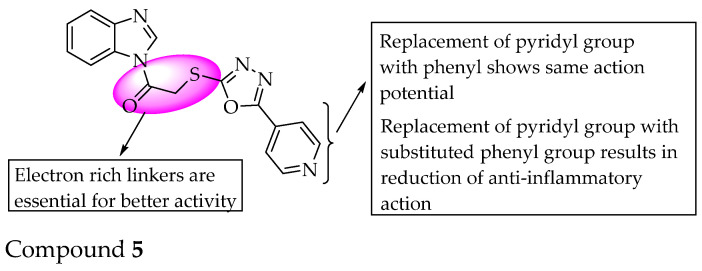
SARs of benzimidazole substituted with oxadiazole.

A study reported that benzimidazole linked to oxadiazole via a thioacetamide linker exhibited strong anti-inflammatory activity (Compound **5**). A reduction in activity was observed when the phenyl ring was substituted with a hydroxy group at the ortho position. Meanwhile, the thioacetamide linker is essential for anti-inflammatory activity [45]. The SARs of the compounds are shown in Figure 27.

**Figure 28:**
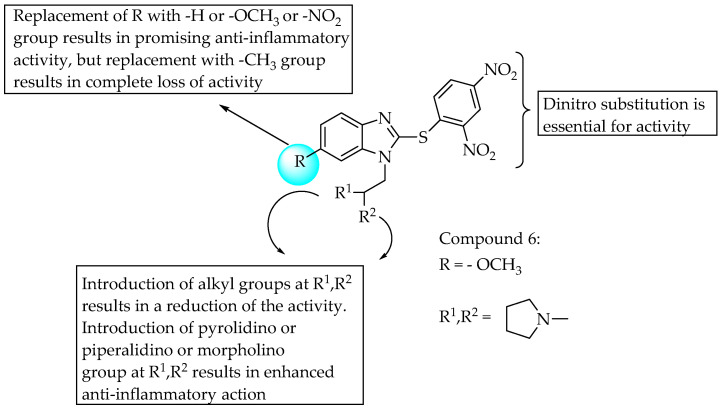
SARs of N-Mannich bases of substituted 2-mercapto-1*H*-benzimidazoles.

Rao et al. (2013) reported anti-inflammatory activities for 20 *N*-Mannich bases of substituted 2-mercapto-1*H*-benzimidazoles using Swiss albino rats as test animals, and the phlogistic agent was 0.1 mL of carrageenan suspension (1% carrageenan in Normal saline). Various substituents in the aromatic ring did not result in much inhibition, but non-substituted benzimidazoles were shown to be inferior to the substituted compounds. A compound bearing an electron-releasing methoxy group at position 6 and a two-pyrrolidine substitution at the nitrogen of benzimidazole (Compound **6**) showed strong anti-inflammatory activity; the percentage inhibition for the paw oedema volume was 43.5. A compound bearing an electron-withdrawing nitro group at the 6-position was more active than the remaining compounds, whereas electron-donating groups led to lower potency [46]. The SARs of the compounds are shown in Figure 28.

**Figure 29:**
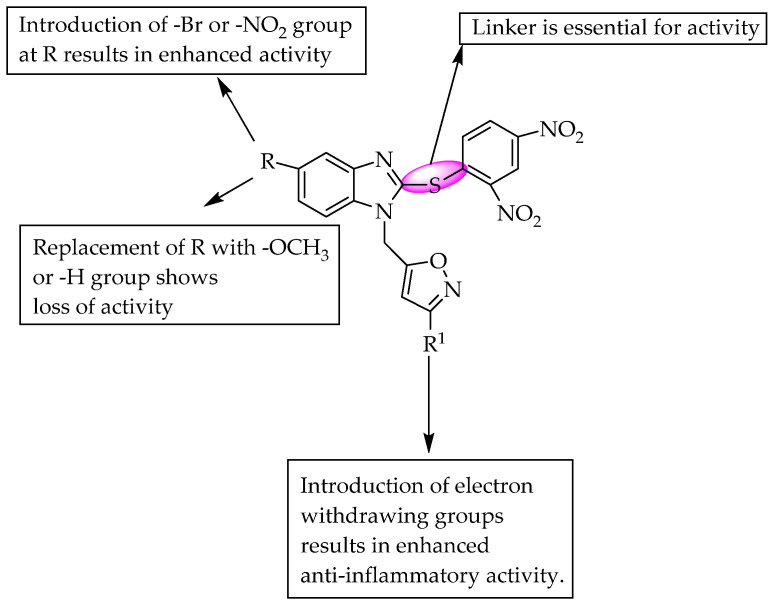
SARs of 3,5-disubstituted isoxazole at N1 of benzimidazole.

Another study by Kankala et al. (2012) explained the synthesis, anti-inflammatory activity and SARs of 3,5-disubstituted isoxazole at the 1-position of benzimidazole. Inflammation and, consequently, oedema in the hind paws of mice were induced using carrageenan at 100 mg/kg body weight. Compounds **7** and **8** possess the electron-withdrawing groups 4-fluorophenyl and 4-cyanophenyl at the C3 position of the isoxazole moiety, which resulted in excellent anti-inflammatory activity, inhibiting the hind paw oedema volume by 60.76 and 58.46%, respectively. However, electronegative groups at C5 of the benzimidazole scaffold resulted in more potent anti-inflammatory activity than the substitution of electron-donating groups or no substitution at the same position [47]. The SARs of the compounds are shown in Figure 29.

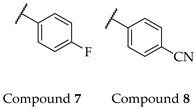



**Figure 30:**
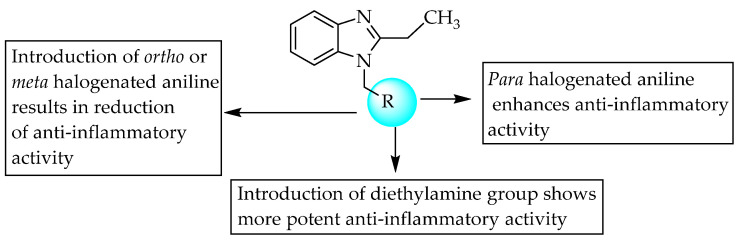
SARs of Mannich bases of 1-(N-substituted amino)methyl-2-ethylbenz–imidazoles.

Mariappan and his co-researchers, in 2011, studied the anti-inflammatory activity of a few Mannich bases of 1-(*N*-substituted amino)methyl-2-ethylbenzimidazoles in mice with carrageenan-induced rat paw oedema. The amount of compound administered was 100 mg/kg p.o. Compounds with 4-fluoro or bromo substituted aniline at R (Figure 30) showed substantial anti-inflammatory effects, with 23 and 25% inhibition of rat paw oedema. Moreover, 2- or 3-halogenated aniline was conducive to anti-inflammatory activity; this may be due to steric hindrance. Overall, electron-withdrawing groups with lipophilicity were preferable for activity [48]. The SARs of the compounds are shown in Figure 30.

**Figure 31:**
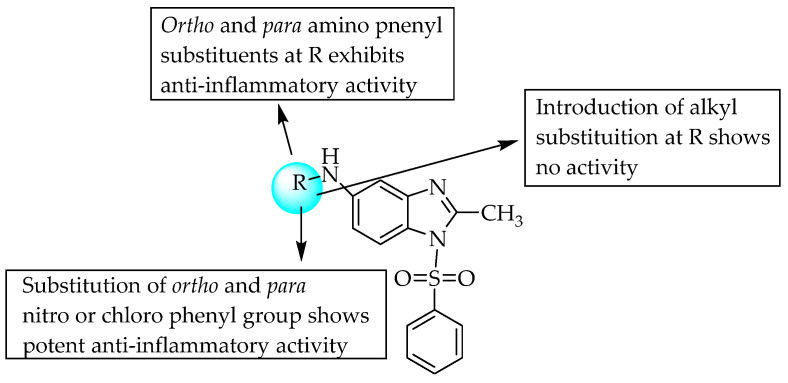
SARs of 5-substituted-1-(phenylsulfonyl)-2-methylbenzimidazoles.

In 2010, Gaba et al. studied the anti-inflammatory activity of 5-substituted-1-(phenylsulfonyl)-2-methyl benzimidazole derivatives using carrageenan-induced paw oedema in a rat model at 200 mg/kg body weight. Compounds substituted with ortho amino (37% reduction) and para amino (39.7% reduction) groups at R (Figure 31) exhibited good anti-inflammatory activity with low ulcerogenic potential due to being neutral molecules bearing –NH_2_. The introduction of –CH_2_ between the amino and aryl moieties of benzimidazole did not influence the anti-inflammatory action [49]. The SARs of the compounds are shown in Figure 31.

**Figure 32:**
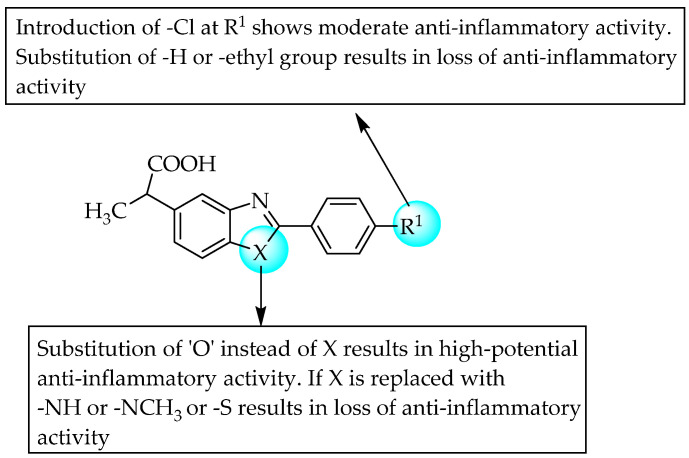
SARs of bio-isosteric replacement of benzoxazole.

**Figure 33:**
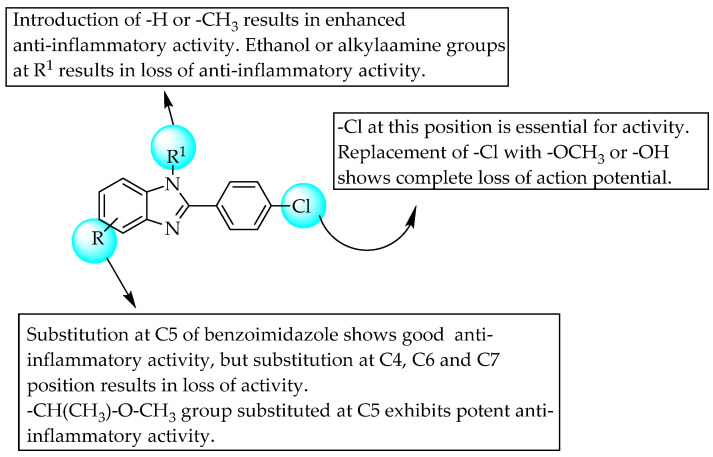
SARs of 4- or 5-position-substituted benzimidazoles.

**Figure 34:**
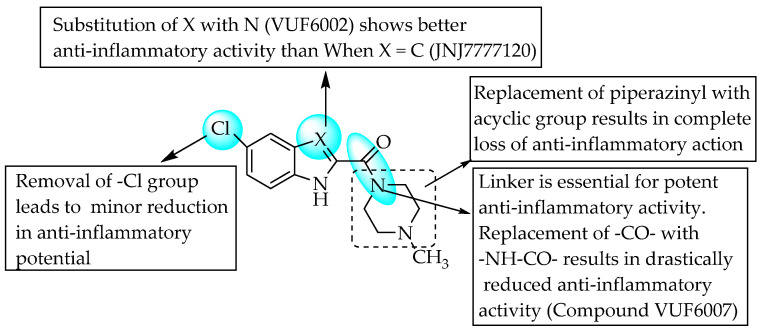
SARs of piperazinyl-substituted benzimidazoles.

Dunwel and his research team tried to evaluate the anti-inflammatory effects of a new series of compounds synthesised by the bio-isosteric replacement of benzoxazole with benzimidazole and benzothiazole, in the context of carrageenan-induced paw oedema in rats. The resulting compounds did not reduce rat paw oedema; this may be due to lower solubility or altered drug–receptor interactions (Figure 32) [50]. Many researchers have tried to develop novel anti-inflammatory agents by introducing substituents simultaneously at C2 and C5 of benzimidazole. Evans et al., in 1996, taking benoxaprofen as a lead, synthesised 72 benzimidazoles and evaluated their anti-inflammatory activities in rat adjuvant arthritis [51]. Only two compounds—a compound with a diethylamino ethoxy moiety at the 6-position and a compound bearing a substitution with 1-methoxy ethyl at C5 of benzimidazole—were comparable to indomethacin, with 43 and 33% improvement, respectively (Figure 33). In 2004, Terzioglu et al. introduced VUF6002 (Figure 34), a potent anti-inflammatory agent with strong antinociceptive effects, using JNJ7777120 (Figure 34) as a lead [52]. 

However, introducing an amide moiety instead of a carbonyl group between the C2 position of benzimidazole (Figure 34) and piperazine resulted in VUF6007, failing to show any such activity. This result conflicts with a few reports indicating the significance of the benzimidazole C2-position amino group [53]. 

**Figure 35:**
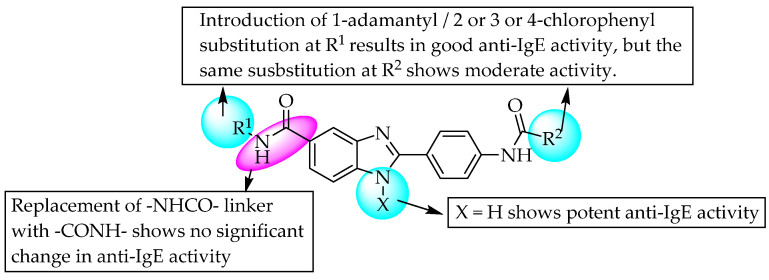
SARs of adamantane-substituted benzimidazoles.

In another study, Avanir Pharmaceuticals developed a compound with potent anti-IgE activity, AVP 13358 [2-(4-adamantanecarboxamido)phenyl)-*N*-(pyridin-2-yl)-1*H*-benzoimidazole-5-carboxamide], bearing an amide linker; IgE plays a role in various inflammatory conditions (Figure 35) [54]. 

**Figure 36:**
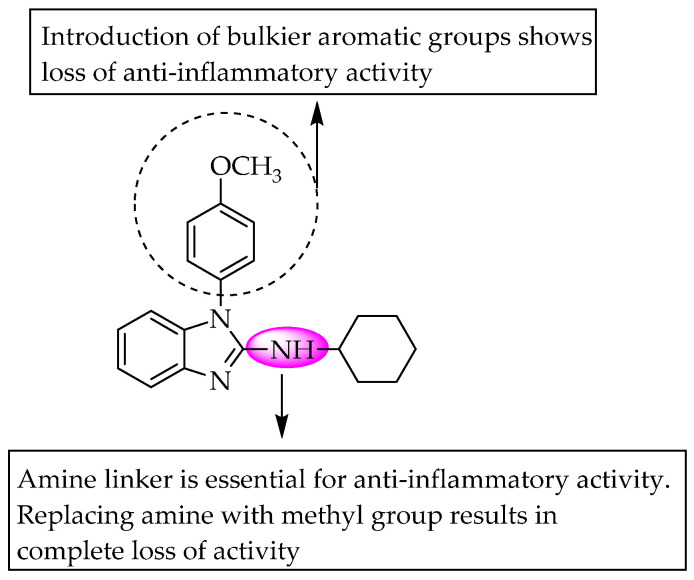
SARs of 1,2-disubstituted benzimidazoles.

The compound 2-cyclohexylamino-1(4-methoxyphenyl)benzimidazole exhibited potent anti-inflammatory activity according to Taniguchi et al. (1993) [55]. The anti-inflammatory activity of the compounds at 100 mg/kg p.o. doses was evaluated using the carrageenan-induced rat paw oedema model. 2-cyclohexylamino-1 (4-methoxy phenyl) benzimidazole exhibited 53.2% inhibition of rat paw oedema. Replacing the amino group with methylene at C2 of benzimidazole significantly reduced the activity, indicating the significance of the guanidine fraction in the activity. Moreover, a bulkier aromatic substitution at the N1 position of benzimidazole was not suitable for activity (Figure 36). Furthermore, they extended their study to designing, synthesising and evaluating the anti-inflammatory activity of 2-iminocycloheptimidazole derivatives and reported that these compounds showed inferior anti-inflammatory action compared to benzimidazole analogues. They concluded that the substitution of hydrophilic groups at the N1 position of cycloheptimidazole resulted in a loss of activity, but 4-methoxyphenyl substitution at the C2 position of cycloheptimidazole did not reduce the anti-inflammatory potential. 

Shen et al. (2010) incorporated the imidazole nucleus with the phenyl ring of an active metabolite isolated from Curvularia verruculo, a synthetic analogue of f152A1. The synthetic analogue had the in vitro inhibitory effect of f152A1 on TNF-α transcription and showed good therapeutic effects in arthritis [56].

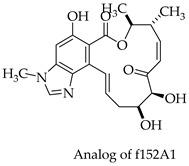



**Figure 37:**
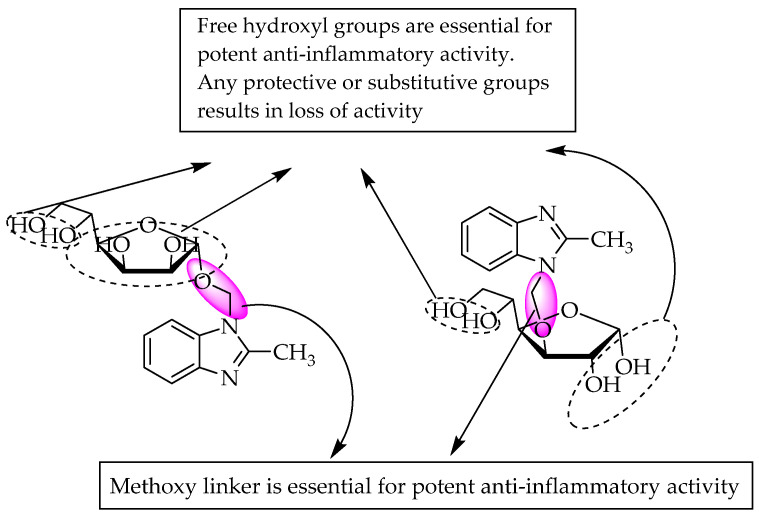
SARs of sugar-linked benzimidazoles.

Conjugated benzimidazoles of 2-methyl-*N*-sugar (Figure 37) were synthesised and evaluated for analgesic and anti-inflammatory activity by El-Nezhawy et al. in 2009 [57]. The anti-inflammatory activity of the compounds at 100 mg/kg p.o. doses was evaluated using the carrageenan-induced rat paw oedema model. The effects of the systemic administration of each of the test drugs at doses of 15, 30 and 60 mg/kg (0.5 mL, i.p., n = 6/group) were studied; they found that the methoxy connector between the N of benzimidazole and the hydroxy group of the sugar was essential for potent anti-inflammatory action. A sugar moiety with free hydroxy groups has been reported to result in greater potency than a protected or derivatised moiety. A sugar connected at its position N3 with benzimidazole was found to result in greater activity (67.8% at 60 mg/kg) than that bridged at position 5 (33.3% at 60 mg/kg) (Figure 37).

**Figure 38:**
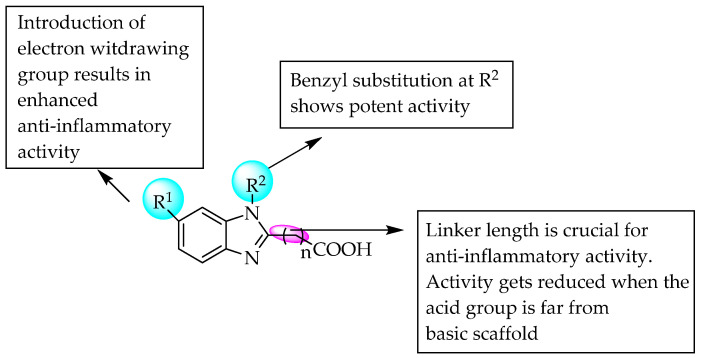
SARs of 1,2,6-trisubstituted benzimidazoles.

A series of 1,2,6-trisubstituted benzimidazoles were synthesised and evaluated for anti-inflammatory activity by Thakurdesai et al. in 2007 [58]. The C2 position was substituted with various carboxylic acids, whereas the C6 position was substituted with electron-rich or poor groups. The activity mainly depends on the groups substituted at C6 of benzimidazole. The activity was inversely related to the length of the linker between the carboxyl group and C2 of benzimidazole (Figure 38). The substitution of the benzyl group at the 1-position enhanced the anti-inflammatory action. 

**Figure 39:**
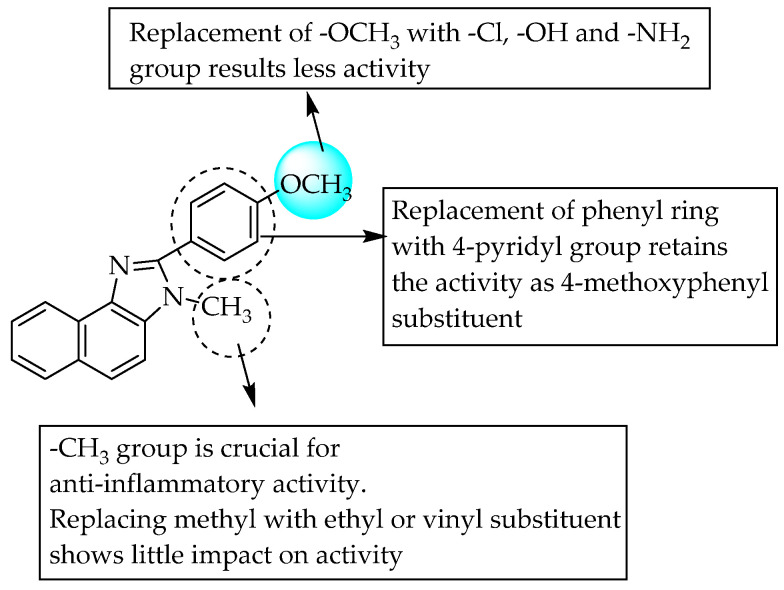
SARs of 1,2-substituted naphthimidazoles.

The anti-inflammatory activity displayed by benzimidazole compounds bearing different substituents has encouraged researchers to concentrate on fused benzimidazoles. Toja et al. (1984) first reported the anti-inflammatory activity of fused imidazole derivatives substituted at the 1- and 2-positions (Figure 39), which were non-acidic agents [59]. The anti-inflammatory activity of the compounds at 100 mg/kg p.o. doses was evaluated using the carrageenan-induced rat paw oedema method. The SARs of the naphthimidazoles revealed that 4-methoxyphenyl substitution at the 2-position of the imidazole nucleus favoured anti-inflammatory activity (45–50% paw oedema inhibition), while a chloro- or hydroxy-substituted phenyl moiety at this position reduced the activity (1–20% paw oedema inhibition).

In another study, benzimidazole–NSAID conjugates were screened for anti-inflammatory activity. The synthesised conjugates matched the parent NSAIDs in potency, while the conjugates with better antioxidant activity notably reduced gastric ulcers. Among the synthesised benzimidazole–NSAID conjugates, benzimidazole–mesalamine showed the most potent anti-inflammatory activity [60].

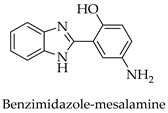



**Figure 40:**
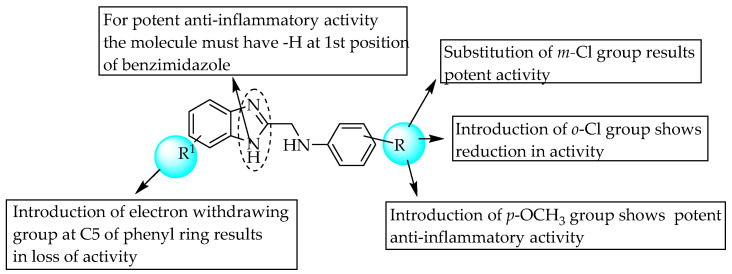
SARs of substituted anilines at C2 position of 6-substituted benzimidazoles.

In 2010, Achar et al. incorporated substituted anilines at the 2-position of 6-substituted benzimidazoles, and screened their anti-inflammatory activity in carrageenan-induced inflammation/oedema in rat hind paws, performing a SAR study (Figure 40). *N*-(1*H*-benzimidazol-2-ylmethyl) aniline and *N*-(1*H*-benzimidazol-2-ylmethyl)-3-chloroaniline showed potent anti-inflammatory (100% at 100 mg/kg) activities compared to nimesulide (100% at 50 mg/kg) [61]. The incorporation of electron-taking groups at the benzimidazole 6-position reduced the anti-inflammatory activity. However, a meta chloro (40–50% paw oedema inhibition) or para methoxy group (30–40% paw oedema inhibition) at R resulted in potent anti-inflammatory activity (Figure 40).

**Figure 41:**
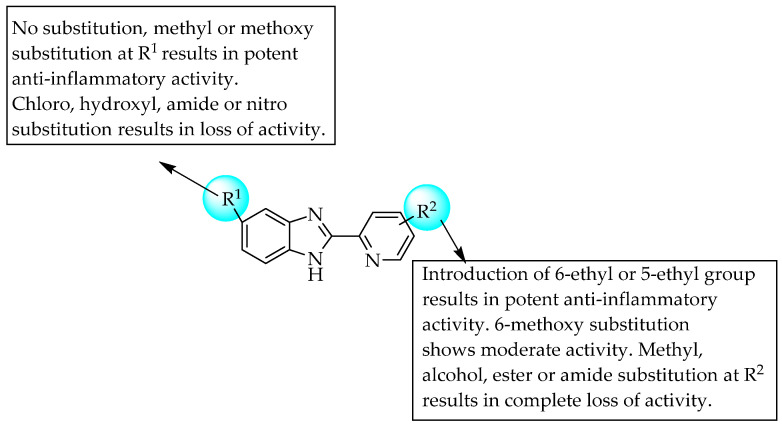
SARs of 2-(2-pyridinyl)benzimidazoles.

The Pharmaceutical Research Centre of Kanebo Ltd. (Japan) developed a few 2-(2-pyridinyl)benzimidazoles by the isosteric replacement of the thiazole ring in thiabendazole, since thiabendazole has moderate anti-inflammatory effects [62,63,64]. They also found that one of the developed compounds, 2-(5-ethyl-2-pyridinyl)benzimidazole (KB-1043), showed better anti-inflammatory activity than phenylbutazone and tiaramide. KB-1043 also showed slightly less gastrointestinal irritation and a 2–3-fold-better therapeutic index than the reference. A compound with a 6-ethyl-2-pyridinyl moiety at the C2 position of benzimidazole also showed comparable activity to KB-1043. However, electron-withdrawing groups at C5 of benzimidazole resulted in a loss of anti-inflammatory activity (Figure 41). 

**Figure 42:**
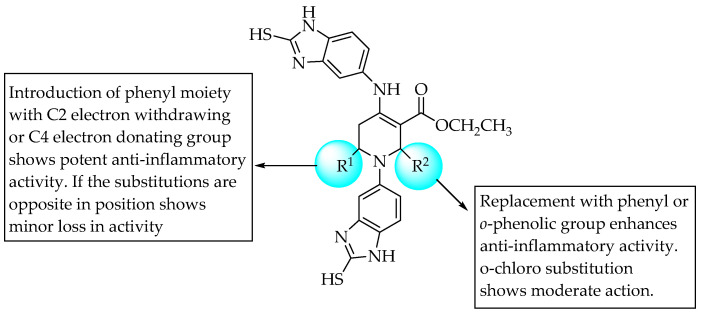
SARs of benzo[d]imidazolyl tetrahydropyridine carboxylates.

Ravindernath and Reddy (2017) reported the anti-inflammatory activity of benzo[d]imidazolyl tetrahydropyridine carboxylates in carrageenan-induced inflammation/oedema in rat hind paws. They found that all the synthesised compounds had moderate anti-inflammatory activity (0.18 to 0.43 at 4 h, volume of oedema (mL)) compared to the standard drug diclofenac sodium (0.60 ± 0.02 at 4 h; control, 3.25 ± 0.03 at 4 h, volume of oedema (mL)). An unsubstituted phenyl or phenyl substituted with 2-position electron-withdrawing groups or 4-position electron-donating groups at R^1^ resulted in potent anti-inflammatory activity. Meanwhile, an ortho phenolic substitution at R^2^ favoured anti-inflammatory activity (Figure 42) [65].

**Figure 43:**
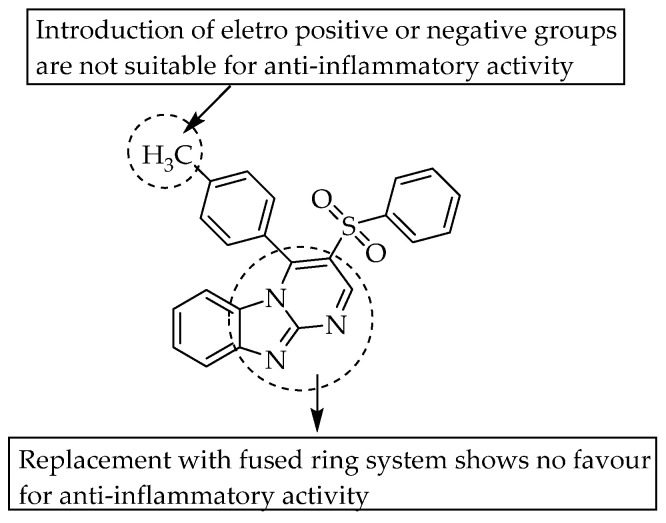
SARs of pyrimido[1,2-a]benzimidazoles.

In another study, a fused pyrimido[1,2-a]benzimidazole with phenylsulfonyl moiety was evaluated for its effect on anti-inflammatory activity. A compound with a fused pyrimido ring exhibited less anti-inflammatory activity than indomethacin (Figure 43) [66]. Both electropositive and negative groups at the phenyl group, which is directly connected to the fused pyrimido moiety, were tested.

**Figure 44:**
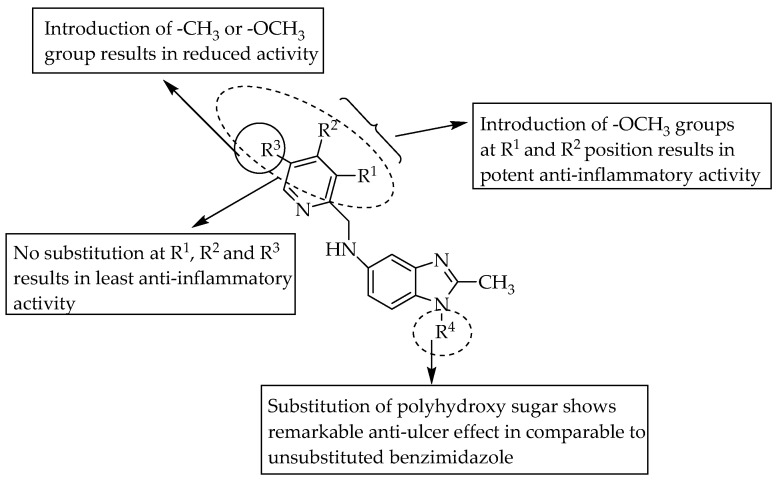
SARs of 5-aminopyridinyl substituted benzimidazoles.

El-Nezhawy et al. (2013) reported the synthesis and anti-inflammatory activity of compounds with a few pyrid-2-yl moieties substituted and polyhydroxy sugar conjugated to the *N*-benzimidazole moiety. The compounds 2-methyl-*N*-((3,4-dimethoxy pyridin-2-yl)methyl)-1*H*-benzimidazol-5-amine and 1-(1,2,3,5-tetrahydroxy-a-d-mannofuranose)-5-(((3,4-dimethoxypyridin-2yl)methyl) amino)-2-methyl-1*H*-benz-imidazole significantly reduced the inflammation in the paw oedema model by 62 and 72%, respectively, which is comparable to the effect of diclofenac (73%). These compounds also showed significant antiulcerogenic activity. They also showed that electron-donating methoxy groups in the pyrid-2-yl moiety mainly contributed to the compounds’ potency. A benzimidazole bearing a polyhydroxy sugar at position one had a potent anti-inflammatory effect, with remarkable anti-ulcer activity (Figure 44) [67].

**Figure 45:**
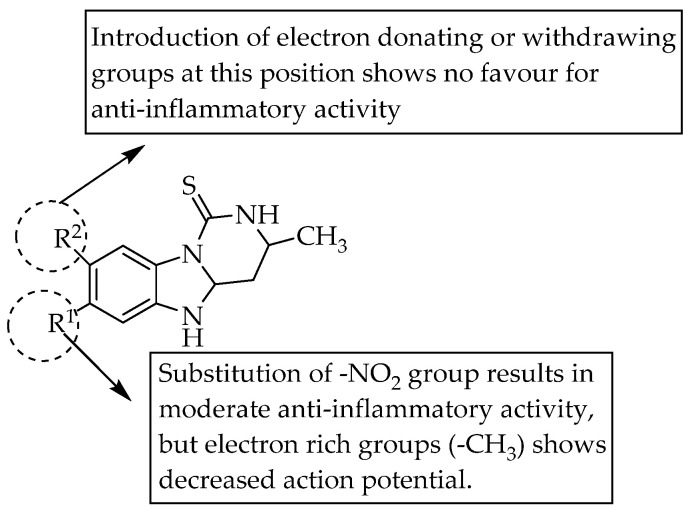
SARs of tetrahydropyrimido[1,6-a]benzimidazol-1(2H)-thione.

Earlier in 2002, Sodhi et al. studied the anti-inflammatory activity of tetrahydropyrimido[1,6-a]benzimidazol-1(2*H*)-thione derivatives with 50 mg/kg p.o. in the model of carrageenan-induced inflammation/oedema in rat hind paws. One of the reported compounds, 3-methyl-8-nitro-3,4,4a,5-tetrahydropyrimido[1,6-a]benzimidazol-1(2*H*)–thione, showed comparable anti-inflammatory activity (46.0, maximum % reduction in oedema) to ibuprofen (51.0, maximum % reduction in oedema). Electron-rich groups at R^1^ and R^2^, and an electron-withdrawing group at R^2^ were unfavourable for anti-inflammatory activity, whereas an –NO_2_ group at R^1^ resulted in moderate anti-inflammatory activity (Figure 45) [68].

**Figure 46:**
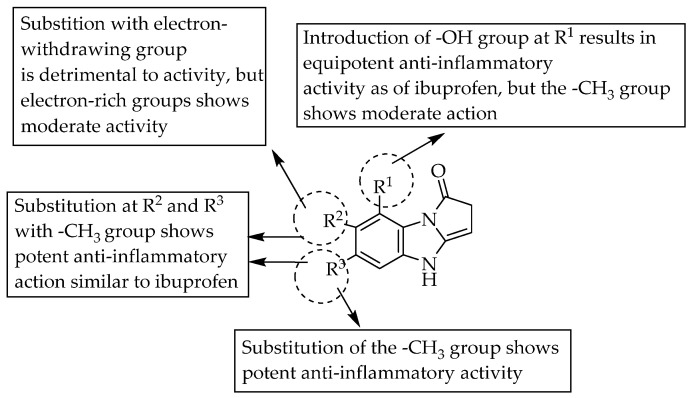
SARs of tricyclic benzimidazoles.

Sondhi et al. (2010) reported the microwave-irradiation synthesis and anti-inflammatory activity of tricyclic benzimidazoles. The anti-inflammatory activity of the compounds was evaluated by using the method of carrageenan-induced inflammation/oedema in rat hind paws. Two of the synthesised compounds, one having a methyl substituent at each position R^2^ and R^3^ (39.4, maximum % reduction in oedema) and another having a hydroxy group at R^1^ (39.2, maximum % reduction in oedema), displayed anti-inflammatory activity comparable to that of ibuprofen (39.0, maximum % reduction in oedema) (Figure 46) [69]. 

Another series of fused benzimidazole ring compounds, 1,2,4-triazolobenzimidazol -3-yl acetohydrazide derivatives, were reported by Mohammed et al. in 2013. The synthesised compounds exhibited significant anti-inflammatory activity, which was evaluated using carrageenan-induced inflammation/oedema in rat hind paws. Two compounds showed potent analgesic and anti-inflammatory (84.2 and 89.3, maximum % reductions in oedema) activity. They also showed GI safety comparable to that of indomethacin (78.8, maximum % reduction in oedema) [70]. 

**Figure 47:**
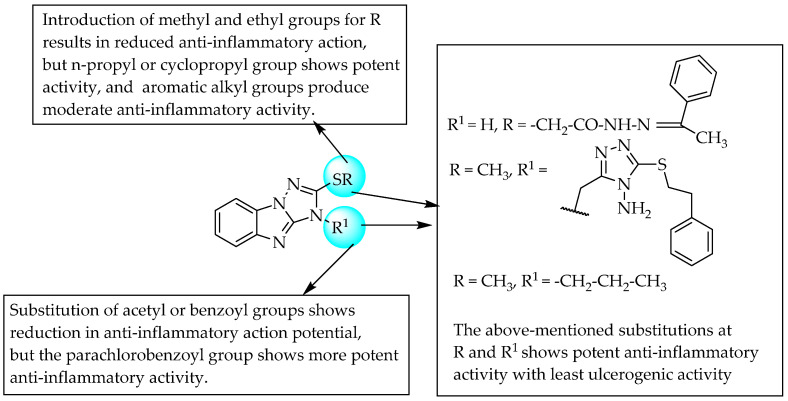
SARs of 1,2,4-triazolobenzimidazoles.

In 2006, the same researchers reported the anti-inflammatory and analgesic activities of a few 1-acyl-2-alkylthio-1,2,4-triazolo [3,2-a]benzimidazole derivatives. A compound containing *N*-acetyl-2-isopropylthio-1,2,4-triazolo [3,2-a]benzimi-dazole exhibited the most potent anti-inflammatory activity. This study highlighted the importance of the amino group at C2 of the benzimidazole nucleus for anti-inflammatory activity (Figure 47) [71]. The compounds showed better GI safety profiles than indomethacin. The SARs of the 1,2,4-triazolobenzimidazoles are depicted in Figure 47.

**Figure 48:**
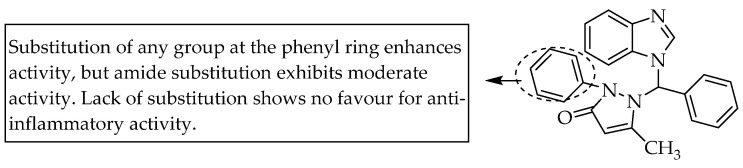
SARs of benzimidazol-1-yl (phenyl)methyl derivatives.

Soni et al. (2011) reported the anti-inflammatory activity of N-substituted benzimidazole derivatives at doses of 200 mg/kg p.o. in the carrageenan-induced model of inflammation/oedema in rat hind paws. One of the synthesised compounds, 1-[1*H*-benzimidazol-1-yl(phenyl)methyl]-5-methyl-2-phenyl-1,2-dihydro-3*H*-pyrazol-3-one, showed a higher log P value and anti-inflammatory activity (75.0, maximum % reduction in oedema) than celecoxib (83.3, maximum % reduction in oedema). As shown in Figure 48, a 2-aryl substitution at the 5-methyl-substituted pyrazol-3-one at the benzimidazole-1-yl methyl moiety enhanced the anti-inflammatory activity, whereas an amide substitution in the same position resulted in moderate activity [72].

**Figure 49:**
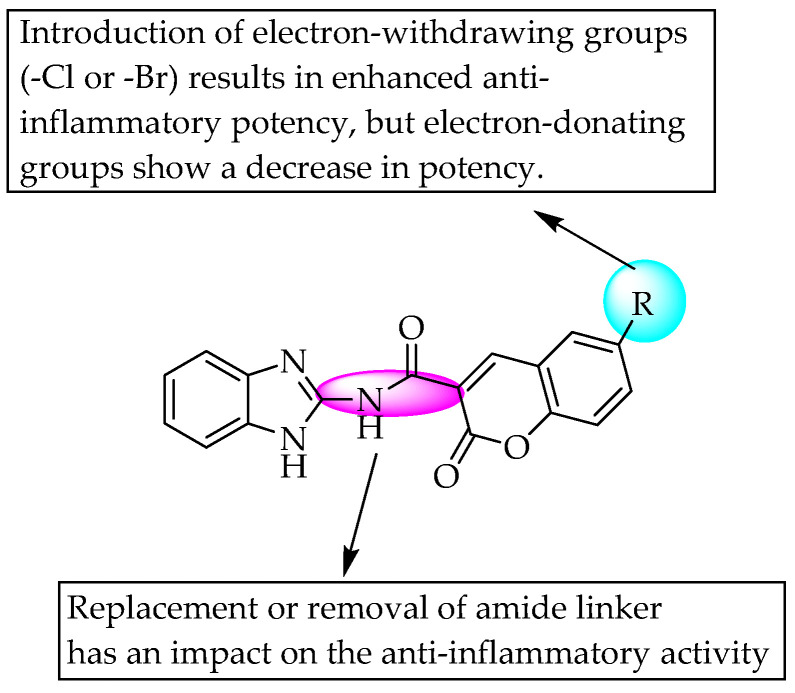
SARs of 2-acetamidobenzimidazoles.

Arora et al. (2014) suggested that electron-deficient groups (–Cl or –Br) favour, whereas electron-rich groups (–OCH_3_) reduce, the anti-inflammatory potency of 2-acetamidobenzimidazoles. They also stated that the amide linkage in the molecule was not favourable for anti-inflammatory activity (Figure 49) [73].

**Figure 50:**
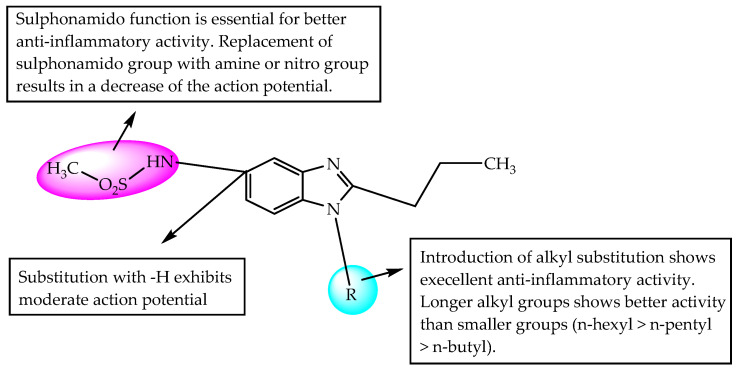
SARs of methanesulphonamido-benzimidazoles.

In another study, Sharma et al. (2017) evaluated methanesulphonamido-benzimidazoles for their gastro-sparing anti-inflammatory effects using a carrageenan-induced model of inflammation/oedema in rat hind paws. Compounds substituted with n-hexyl, n-pentyl and n-butyl at N1 of benzimidazole showed better anti-inflammatory activity (92.73, 95.64 and 97.62, maximum % reduction in oedema) than rofecoxib and indomethacin (78.95 and 75.00, maximum % reduction in oedema). All the synthesised compounds were non-ulcerogenic at the tested doses. The methane-sulphonamido substitution at C5 of benzimidazole is essential for potent anti-inflammatory activity (Figure 50) [74].

## 4. Summary and Perspectives

**Figure 51:**
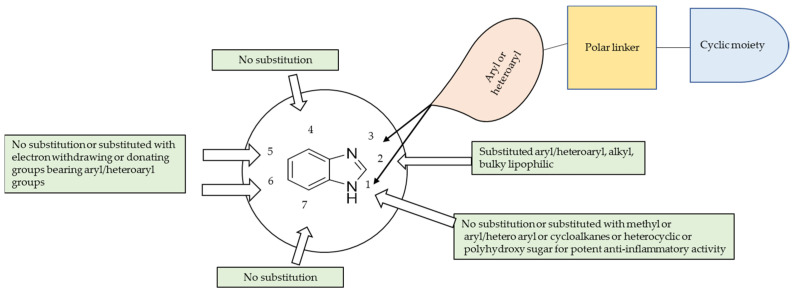
Summary of substituents at benzimidazole nucleus favourable for potent anti-inflammatory activity.

The substituent groups in benzimidazole favoured for potent anti-inflammatory activity are shown in Figure 51. For developing benzimidazoles as anti-inflammatory agents, substitutions at N1, C2, C5 and C6 were preferable. Specifically, an aryl/heteroaryl substitution at C5 or C6 enhances the anti-inflammatory activity, while being unsubstituted at C4 and C7 is better for anti-inflammatory activity. However, an amine group at C2 consistently enhanced the anti-inflammatory activity, while a methyl or ethyl linker between the N1 of benzimidazole and the substituent groups was essential for potent anti-inflammatory activity. 

Worldwide, NSAIDs have been successfully used to relieve pain and inflammation and continue to be used every day by thousands of people. There has been tremendous growth in the last two to three decades in the design and development of anti-inflammatory agents. However, safe and effective therapy for inflammatory conditions remains challenging in many ways. The chronic use of NSAIDs is linked to adverse effects on cardiovascular health. Therefore, a benzimidazole scaffold-containing compound may be used since it possesses analgesic and anti-inflammatory action along with angiotensin II-receptor-blocking activity. Benzimidazole derivatives act through different mechanisms, such as reducing cytokines, TRPV-1 antagonism, cannabinoid receptor agonism and FLAP inhibition. Hence, there is a possibility that a single benzimidazole derivative can be optimised to act through multiple pathways involved in pain and inflammation.

This approach may provide benefits over the use of combinations of analgesic anti-inflammatory drugs, with different mechanisms and fewer side effects. A benzimidazole scaffold may be the best pharmacophore for designing active small molecules with good anti-inflammatory effects based on the above-mentioned unique properties.

## 5. Conclusions

Benzimidazole is a vital scaffold in medicinal chemistry because of its diverse biological activities. Benzimidazoles act via different mechanisms in treating numerous diseases, as discussed in the Introduction of this review. Omeprazole, lansoprazole, pantoprazole, albendazole, mebendazole, thiabendazole, astemizole, enviradene, candesartan, cilexitil and telmisartan are clinically approved drugs that contain benzimidazole nuclei. Several researchers have explored the synthesis, structure–activity relationships, QSARs, molecular modelling and other physicochemical and pharmacokinetic profiles of benzimidazoles. In our view, a complete understanding of the structural, physical and chemical properties of the benzimidazoles may help researchers to better determine their potential use in treating inflammation. However, some severe side effects associated with benzimidazoles can be identified and need to be rectified. Further research in this field is need using advanced techniques, such as QSAR analysis, pharmacophore mapping and docking studies, and known SAR information reported in the literature will bring about novel benzimidazoles with considerable scope for use. These methods can provide a much more direct picture of the structural features contributing to the SARs of benzimidazoles.

## Figures and Tables

**Table 1:**
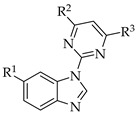
N1-substituted benzimidazoles with pyrimidin-2-yl.

	Compound 2	Compound 3
R^1^	H	CN
R^2^	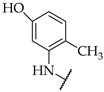	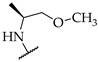
R^3^	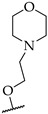	H

## Data Availability

Data sharing not applicable.

## References

[1] Hansson G.K., Libby P. (2006). The Immune Response in Atherosclerosis: A Double-Edged Sword. Nat. Rev. Immunol..

[2] Libby P. (2002). Atherosclerosis: The New View. Sci. Am..

[3] Medzhitov R. (2010). Inflammation 2010: New Adventures of an Old Flame. Cell.

[4] Takeuchi O., Akira S. (2010). Pattern Recognition Receptors and Inflammation. Cell.

[5] Poetker D.M., Reh D.D. (2010). A Comprehensive Review of the Adverse Effects of Systemic Corticosteroids. Otolaryngol. Clin..

[6] Harirforoosh S., Asghar W., Jamali F. (2013). Adverse Effects of Nonsteroidal Antiinflammatory Drugs: An Update of Gastrointestinal, Cardiovascular and Renal Complications. J. Pharm. Pharm. Sci..

[7] Day R.O., Graham G.G. (2013). Non-Steroidal Anti-Inflammatory Drugs (NSAIDs). BMJ.

[8] Domanskyi A., Geißler C., Vinnikov I.A., Alter H., Schober A., Vogt M.A., Gass P., Parlato R., Schütz G. (2011). Pten Ablation in Adult Dopaminergic Neurons Is Neuroprotective in Parkinson’s Disease Models. FASEB J..

[9] Kaur G., Silakari O. (2018). Benzimidazole Scaffold Based Hybrid Molecules for Various Inflammatory Targets: Synthesis and Evaluation. Bioorg. Chem..

[10] Kumar B., Kumar R., Skvortsova I., Kumar V. (2017). Mechanisms of Tubulin Binding Ligands to Target Cancer Cells: Updates on Their Therapeutic Potential and Clinical Trials. Curr. Cancer Drug Targets.

[11] Dhiman P., Arora N., Thanikachalam P.V., Monga V. (2019). Recent Advances in the Synthetic and Medicinal Perspective of Quinolones: A Review. Bioorg. Chem..

[12] Sharma S., Kumar D., Singh G., Monga V., Kumar B. (2020). Recent Advancements in the Development of Heterocyclic Anti-Inflammatory Agents. Eur. J. Med. Chem..

[13] Narasimhan B., Sharma D., Kumar P. (2012). Benzimidazole: A Medicinally Important Heterocyclic Moiety. Med. Chem. Res..

[14] Bansal Y., Silakari O. (2012). The Therapeutic Journey of Benzimidazoles: A Review. Bioorg. Med. Chem..

[15] Ingle R.G., Magar D.D. (2011). Heterocyclic Chemistry of Benzimidazoles and Potential Activities of Derivatives. Int. J. Drug Res. Technol..

[16] Paramashivappa R., Kumar P.P., Rao P.V.S., Rao A.S. (2003). Design, Synthesis and Biological Evaluation of Benzimidazole/ Benzothiazole and Benzoxazole Derivatives as Cyclooxygenase Inhibitors Bioorg. Med. Chem. Lett..

[17] Bukhari S.N.A., Lauro G., Jantan I., Fei Chee C., Amjad M.W., Bifulco G., Sher H., Abdullah I., Rahman N.A. (2016). Antiinflammatory Trends of New Benzimidazole Derivatives. Future Med. Chem..

[18] Cheng Y., Hitchcock S.A. (2007). Targeting Cannabinoid Agonists for Inflammatory and Neuropathic Pain. Expert Opin. Investig. Drugs.

[19] Watson C., Owen D.R., Harding D., Kon-I K., Lewis M.L., Mason H.J., Matsumizu M., Mukaiyama T., Rodriguez-Lens M., Shima A. (2011). Optimisation of a Novel Series of Selective CNS Penetrant CB2 Agonists. Bioorg. Med. Chem. Lett..

[20] Gijsen H.J.M., Cleyn M.A.J.D., Surkyn M., Van Lommen G.R.E., Verbist B.M.P., Nijsen M.J.M.A., Meert T., Wauwe J.V., Aerssens J. (2012). 5-Sulfonyl-Benzimidazoles as Selective CB2 Agonists-Part 2. Bioorg. Med. Chem. Lett..

[21] Sondhi S.M., Singh N., Kumar A., Lozach O., Meijer L. (2006). Synthesis, Anti-Inflammatory, Analgesic and Kinase (CDK-1, CDK-5 and GSK-3) Inhibition Activity Evaluation of Benzimidazole/Benzoxazole Derivatives and Some Schiff’s Bases. Bioorg. Med. Chem..

[22] Dombroski M.A., Letavic M.A., McClure K.F. (2002). Benzimidazole Anti-Inflammatory Compounds. U.S. Patent.

[23] Anderskewitz R., Birke F., Bouyssou T., Dollinger H., Martyres D., Pouzet P. (2007). Haloalkyl-and Piperidine-Substituted Benzimidazole-Derivatives. U.S. Patent.

[24] Mader M., de Dios A., Shih C., Bonjouklian R., Li T., White W., López de Uralde B., Sánchez-Martinez C., del Prado M., Jaramillo C. (2008). Imidazolyl Benzimidazoles and Imidazo[4,5-b]Pyridines as Potent P38alpha MAP Kinase Inhibitors with Excellent in Vivo Antiinflammatory Properties. Bioorg. Med. Chem. Lett..

[25] Guo Q., Chandrasekhar J., Ihle D., Wustrow D.J., Chenard B.L., Krause J.E., Hutchison A., Alderman D., Cheng C., Cortright D. (2008). 1-Benzylbenzimidazoles: The Discovery of a Novel Series of Bradykinin B1 Receptor Antagonists. Bioorg. Med. Chem. Lett..

[26] Zischinsky G., Stragies R., Schaudt M., Pfeifer J.R., Gibson C., Locardi E., Scharn D., Richter U., Kalkhof H., Dinkel K. (2010). Novel Small Molecule Bradykinin B1 Receptor Antagonists. Part 2: 5-Membered Diaminoheterocycles. Bioorg. Med. Chem. Lett..

[27] Dios A.D., Shih C.B., Uralde L.D., Sanchez C., Prado M.D., Cabrejas M.M. (2005). Design of Potent and Selective 2-Aminobenzimidazole-Based P38a MAP Kinase Inhibitors with Excellent in Vivo Efficacy. J. Med. Chem..

[28] Powers J.P., Li S., Jaen J.C., Liu J., Walker N.P.C., Wang Z., Wesche H. (2006). Discovery and Initial SAR of Inhibitors of Interleukin-1 Receptor-Associated Kinase-4. Bioorg. Med. Chem. Lett..

[29] Frenkel A.D., Lively S.E., Powers J.P., Smith A., Sun D., Tomooka C., Wang Z. (2010). Benzimidazole Derivatives. U.S. Patent.

[30] Hayes M.E., Wallace G.A., Grongsaard P., Bischoff A., George D.M., Miao W., McPherson M.J., Stoffel R.H., Green D.W., Roth G.P. (2008). Discovery of Small Molecule Benzimidazole Antagonists of the Chemokine Receptor CXCR3. Bioorg. Med. Chem. Lett..

[31] Sabat M., VanRens J.C., Laufersweiler M.J., Brugel T.A., Maier J., Golebiowski A., De B., Easwaran V., Hsieh L.C., Walter R.L. (2006). The Development of 2-Benzimidazole Substituted Pyrimidine Based Inhibitors of Lymphocyte Specific Kinase (Lck). Bioorg. Med. Chem. Lett..

[32] Chen J.J., Thakur K.D., Clark M.P., Laughlin S.K., George K.M., Bookland R.G., Davis J.R., Cabrera E.J., Easwaran V., De B. (2006). Development of Pyrimidine-Based Inhibitors of Janus Tyrosine Kinase 3. Bioorg. Med. Chem. Lett..

[33] Martin M.W., Newcomb J., Nunes J.J., Boucher C., Chai L., Epstein L.F., Faust T., Flores S., Gallant P., Gore A. (2008). Structure-Based Design of Novel 2-Amino-6-Phenylpyrimido[50,40:5,6]Pyrimido[1,2-a]Benzimidazol-5(6H)-Ones as Potent and Orally Active Inhibitors of Lymphocyte Specific Kinase (Lck): Synthesis, SAR and in Vivo Anti-Inflammatory Activity. J. Med. Chem..

[34] Hunt J.A., Beresis R.T., Goulet J.L., Holmes M.A., Hong X.J., Kovacs E., Mills S.G., Ruzek R.D., Wong F., Hermes J.D. (2009). Disubstituted Pyrimidines as Lck Inhibitors. Bioorg. Med. Chem. Lett..

[35] Banoglu E., Çalişkan B., Luderer S., Eren G., Özkan Y., Altenhofen W., Weinigel C., Barz D., Gerstmeier J., Pergola C. (2012). Identification of Novel Benzimidazole Derivatives as Inhibitors of Leukotriene Biosynthesis by Virtual Screening Targeting 5-Lipoxygenase-Activating Protein (FLAP). Bioorg. Med. Chem..

[36] Evans J.F., Ferguson A.D., Mosley R.T., Hutchinson J.H. (2008). What’s All the FLAP about? 5-Lipoxygenase-Activating Protein Inhibitors for Inflammatory Diseases. Trends Pharmacol. Sci..

[37] Hutchinson H., Li Y., Arruda J.M., Baccei C., Bain G., Chapman C., Correa L., Darlington J., King C.D., Lee C. (2009). 5-Lipoxygenase-Activating Protein Inhibitors: Development of 3-[3-Tert-Butylsulfanyl-1-[4-(6-Methoxy-Pyridin-3-Yl)- Benzyl]-5-(Pyridin-2-Ylmethoxy)-1H-Indol-2-Yl] -2,2-Dimethyl-Propionic Acid (AM103). J. Med. Chem..

[38] Stock N., Baccei C., Bain G., Chapman C., Correa L., Darlington J., King C., Lee C., Lorrain D.S., Prodanovich P. (2010). 5-Lipoxygenase-Activating Protein Inhibitors. Part 3: 3-{3-Tert- Butylsulfanyl-1-[4-(5-Methoxy-Pyrimidin-2-Yl)-Benzyl]-5-(5-Methyl-Pyridin-2-Ylmethoxy)-1H-Indol-2-Yl]-2,2-Dimethyl-Propionic Acid (AM643)-A Potent FLAP Inhibitor Suitable for Topical Administration. Bioorg. Med. Chem. Lett..

[39] Stock N., Baccei C., Bain G., Broadhead A., Chapman C., Darlington J., King C., Lee C., Li Y., Lorrain D.S. (2010). 5-Lipoxygenase-Activating Protein Inhibitors. Part 2: 3-{5-((S)-1-Acetyl-2,3-Dihydro-1H-Indol-2-Ylmethoxy)-3-Tert-Butylsulfanyl-1-[4-(5-Methoxy-Pyrimidin-2-Yl)-Benzyl]-1H-Indol-2-Yl}-2,2-Dimethyl-Propionic Acid (AM679)-a Potent FLAP Inhibitor. Bioorg. Med. Chem. Lett..

[40] Stock N.S., Bain G., Zunic J., Li Y., Ziff J., Roppe J., Santini A., Darlington J., Prodanovich P., King C.D. (2011). 5-Lipoxygenase activating Protein (FLAP) Inhibitors. Part 4: Development of 3-[3-Tert-Butylsulfanyl- 1-[4-(6-Ethoxypyridin-3-Yl) Benzyl]-5-(5-Methylpyridin-2- Ylmethoxy)-1H-Indol-2-Yl]-2,2-Dimethylpropionic Acid (AM803), a Potent, Oral, Once Daily FLAP Inhibitor. J. Med. Chem..

[41] Ognyanov V.I., Balan C., Bannon A.W., Bo Y., Dominguez C., Fotsch C., Gore V.K., Klionsky L., Ma V.V., Qian Y.X. (2006). Design of Potent, Orally Available Antagonists of the Transient Receptor Potential Vanilloid 1. Structureeactivity Relationships of 2-Piperazin-1-Yl-1H Benzimidazoles. J. Med. Chem..

[42] Rami H.K., Gunthorpe M.J. (2004). The Therapeutic Potential of TRPV1 (VR1) Antagonists: Clinical Answers Await. Drug Discov. Today Ther. Strateg..

[43] Fletcher S.R., McIver E., Lewis S., Burkamp F., Leech C., Mason G., Boyce S., Morrison D., Richards G., Sutton K. (2006). The Search for Novel TRPV1- Antagonists: From Carboxamides to Benzimidazoles and Indazolones. Bioorg. Med. Chem. Lett..

[44] Bamborough P., Christopher J.A., Cutler G.J., Dickson M.C., Mellor G.W., Morey J.V., Patel C.B., Shewchuk L.M. (2006). 5-(1H-Benzimidazol-1-Yl)-3-Alkoxy-2-Thiophenecarbonitriles as Potent, Selective, Inhibitors of IKK-Epsilon Kinase. Bioorg. Med. Chem. Lett..

[45] Rajasekaran S., Rao G., Chatterjee A. (2012). Synthesis, Anti-Inflammatory and Antioxidant Activity of Some Substituted Benzimidazole Derivatives. Int. J. Drug Dev. Res..

[46] Rao G.M., Reddy Y.N., Kumar B.V. (2013). Evaluation of Analgesic and Antiinflammatory Activities of N-Mannich Bases of Substituted 2-Mercapto-1Hbenzimidazoles. Int. J. Appl. Biol. Pharm. Technol..

[47] Kankala S., Kankala R.K., Gundepaka P., Thota N., Nerella S., Gangula M.R., Guguloth H., Kagga M., Vadde R., Vasam C.S. (2013). Regioselective Synthesis of Isoxazole-Mercapto Benzimidazole Hybrids and Their in Vivo Analgesic and Anti-Inflammatory Activity Studies. Bioorg. Med. Chem. Lett..

[48] Mariappan G., Bhuyan N.R., Kumar P., Kumar D., Murali K. (2011). Synthesis and Biological Evaluation of Mannich Bases of Benzimidazole Derivatives. Ind. J. Chem..

[49] Gaba M., Singh D., Singh S., Sharma V., Gaba P. (2010). Synthesis and Pharmacological Evaluation of Novel 5-Substituted-1- (Phenylsulfonyl)-2-Methylbenzimidazole Derivatives as Anti-Inflammatory and Analgesic Agents. Eur. J. Med. Chem..

[50] Dunwell D.W., Evans D., Smith C.E., Williamson W.R. (1975). Synthesis and Antiinflammatory Activity of Some-2-Substituted Alpha-Methyl-5-Benzimidazoleacetic Acids. J. Med. Chem..

[51] Evans D., Hicks T.A., Williamson W.R.N., Dawson W., Meacock S.C.R., Kitchen E.A. (1996). Synthesis of a Group of 1H--Benzimidazoles and Their Screening for Antiinflammatory Activity. Eur. J. Med. Chem..

[52] Terzioglu N., van Rijn R.M., Bakker R.A., De Esch I.J.P., Leurs R. (2004). Synthesis and Structure-Activity Relationships of Indole and Benzimidazole Piperazines as Histamine H(4) Receptor Antagonists. Bioorg. Med. Chem. Lett..

[53] Coruzzi G., Adami M., Guaita E., de Esch I.J.P., Leurs R. (2007). Antiinflammatory and Antinociceptive Effects of the Selective Histamine H4-Receptor Antagonists JNJ7777120 and VUF6002 in a Rat Model of Carrageenan-Induced Acute Inflammation. Eur. J. Pharmacol..

[54] Richards M.L., Lio S.C., Sinha A., Banie H., Thomas R.J., Major M., Tanji M., Sircar J.C. (2006). Substituted 2-Phenyl- Benzimidazole Derivatives: Novel Compounds That Suppress Key Markers of Allergy. Eur. J. Med. Chem..

[55] Taniguchi K., Shigenaga S., Ogahara T., Fujitsu T., Matsuo M. (1993). Synthesis and Anti- Inflammatory and Analgesic Properties of 2-Amino-1H-Benzimidazole and 1,2-Dihydro-2- Iminocycloheptimidazole Derivatives. Chem. Pharm. Bull..

[56] Shen Y., Boivin R., Yoneda N., Du H., Schiller S., Matsushima T., Goto M., Shirota H., Gusovsky F., Lemelin C. (2010). Discovery of Antiinflammatory Clinical Candidate E6201, Inspired from Resorcylic Lactone LLZ1640-2, III. Bioorg. Med. Chem. Lett..

[57] El-Nezhawy A.O.H., Gaballah S.T., Radwan M.A.A., Baiuomy A.R., Abdel-Salam O.M. (2009). Structure-Based Design of Benzimidazole Sugar Conjugates: Synthesis, SAR and in Vivo Anti-Inflammatory and Analgesic Activities. Med. Chem..

[58] Thakurdesai P.A., Wadodkar S.G., Chopade C.T. (2007). Synthesis and Anti-Inflammatory Activity of Some Benzimidazole-2- Carboxylic Acids. Pharmacologyonline.

[59] Toja E., Selva D., Schiatti P. (1984). 3-Alkyl-2-Aryl-3H-Naphth[1,2-d]Imidazoles, a Novel Class of Non-acidic Anti-inflammatory Agents. J. Med. Chem..

[60] Bansal Y., Silakari O. (2014). Synthesis and Pharmacological Evaluation of Polyfunctional Benzimidazole-NSAID Chimeric Molecules Combining Antiinflammatory, Immunomodulatory and Antioxidant Activities. Arch. Pharm. Res..

[61] Achar K.C.S., Hosamani K.M., Seetharamareddy H.R. (2010). In-Vivo Analgesic and Anti-Inflammatory Activities of Newly Synthesized Benzimidazole Derivatives. Eur. J. Med. Chem..

[62] Tsukamoto G., Yoshino K., Kohno T., Ohtaka H., Kagaya H., Ito K. (1980). 2-Substituted Azole Derivatives. 1. Synthesis and Anti-inflammatory Activity of Some 2-(Substituted-Pyridinyl)Benzimidazoles. J. Med. Chem..

[63] Ito K., Kagaya H., Fukuda T., Yoshino K., Nose T. (1982). Pharmacological Studies of a New Non-Steroidal Antiinflammatory Drug: 2-(5-Ethylpyridin-2-Yl)Benzimidazole (KB-1043). Arzneimittel-forschung.

[64] Ito K., Kagaya H., Satoh I., Tsukamoto G., Nose T. (1982). The Studies of the Mechanism of Antiinflammatory Action of 2-(5-Ethylpyridin-2-Yl)Benzimidazole (KB-1043). Arzneimittel-forschung.

[65] Ravindernath A., Reddy M.S. (2017). Synthesis and Evaluation of Anti-Inflammatory, Antioxidant and Anti-microbial Activities of Densely Functionalised Novel Benzo [d] Imidazolyl Tetrahydropyridine Carboxylates. Arab. J. Chem..

[66] Shaaban M.R., Saleh T.S., Mayhoub A.S., Mansour A., Farag A.M. (2008). Synthesis and Analgesic/Anti-Inflammatory Evaluation of Fused Heterocyclic Ring Systems Incorporating Phenylsulfonyl Moiety. Bioorg. Med. Chem..

[67] El-Nezhawy A.O., Biuomy A.R., Hassan F.S., Ismaiel A.K., Omar H.A. (2013). Design Synthesis and Pharmacological Evaluation of Omeprazole-like Agents with Anti-inflammatory Activity. Bioorg. Med. Chem..

[68] Sondhi S.M., Rajvanshi S., Johar M., Bharti N., Azam A., Singh A.K. (2002). Anti-Inflammatory, Analgesic and Antiamoebic Activity Evaluation of Pyrimido[1,6-a] Benzimidazole Derivatives Synthesised by the Reaction of Ketoisothiocyanates with Mono and Diamines. Eur. J. Med. Chem..

[69] Sondhi S.M., Rani R., Singh J., Roy P., Agrawal S.K., Saxena A.K. (2010). Solvent Free Synthesis, Anti-Inflammatory and Anticancer Activity Evaluation of Tricyclic and Tetracyclic Benzimidazole Derivatives. Bioorg. Med. Chem. Lett..

[70] Mohammed A.F., Abdel-Moty S.G., Hussein M.A., Abdel-Alim A.M. (2013). Design, Synthesis and Molecular Docking of Some New 1,2,4-Triazolobenzimidazol-3-Yl Acetohydrazide Derivatives with Anti-Inflammatory Analgesic Activities. Arch. Pharm. Res..

[71] Mohamed B.G., Abdel-Alim M., Mostafa A.H. (2006). Synthesis of 1-Acyl-2-Alkylthio-1,2,4-Triazolobenzimidazoles with Anti-fungal, Anti-Inflammatory and Analgesic Effects. Acta Pharm..

[72] Soni J.P., Sen D.J., Modh K.M. (2011). Structure Activity Relationship Studies of Synthesised Pyrazolone Derivatives of Imidazole, Benzimidazole and Benztriazole Moiety for Anti-Inflammatory Activity. J. Appl. Pharm. Sci..

[73] Arora R.K., Kaur N., Bansal Y., Bansal G. (2014). Novel Coumarin-Benzimidazole Derivatives as Antioxidants and Safer Anti-Inflammatory Agents. Acta Pharm. Sin. B.

[74] Sharma R., Bali A., Chaudhari B.B. (2017). Synthesis of Methanesulphonamido-Benzimidazole Derivatives as Gastro-Sparing Anti-inflammatory Agents with Antioxidant Effect. Bioorg. Med. Chem. Lett..

